# SARS-CoV-2 infection in female sex workers from Nairobi, Kenya early in the COVID-19 pandemic: Seroincidence and behavioural associations

**DOI:** 10.1371/journal.pone.0327692

**Published:** 2026-06-15

**Authors:** Su D. Yang, Freda Qi, Karen Colwill, Anne-Claude Gingras, Tara S. Beattie, Joshua Kimani, Rupert Kaul

**Affiliations:** 1 Department of Immunology, University of Toronto, Toronto, Canada; 2 Lunenfeld-Tanenbaum Research Institute at Mount Sinai Hospital Sinai Health Toronto, Canada; 3 Department of Molecular Genetics, University of Toronto, Toronto, Canada; 4 Department of Global Health and Development, London School of Hygiene and Tropical Medicine, London, United Kingdom; 5 Partners for Health and Development in Africa, Nairobi, Kenya; 6 Department of Medicine, University of Toronto, Toronto, Canada; De Montfort University Faculty of Health and Life Sciences, UNITED KINGDOM OF GREAT BRITAIN AND NORTHERN IRELAND

## Abstract

While COVID-19 mortality was relatively low in many Sub-Saharan African countries during the first wave of the pandemic, SARS-CoV-2 transmission was extensive. We hypothesized that female sex workers (FSWs) would be at enhanced risk of acquisition of this novel respiratory viral infection, due to intimate contact with multiple sexual partners and solicitation of clients in crowded venues. Here we describe the seroincidence, socio-behavioural associations and clinical outcomes of SARS-CoV-2 infection in Kenyan FSWs early in the pandemic. A longitudinal cohort of 1003 FSWs (257 living with HIV) from Nairobi, Kenya was enrolled in mid-2019, just prior to the pandemic, and plasma was available for SARS-CoV-2 serology from 827 participants at clinical follow up approximately one year later. Socio-behavioural and respiratory symptom data were collected by questionnaire. We examined the association of SARS-CoV-2 infection with socio-behavioural factors. Follow-up was a median of 201 days (range; 92−342 days) after the declaration of the COVID-19 pandemic in Kenya, and 229 (27.7%) participants were SARS-CoV-2 seropositive. Seroprevalence increased steadily with time from pandemic declaration. Infection was not associated with behavioural or demographic parameters but was strongly associated with time since start of the pandemic (p < 0.0001). Respiratory symptoms during the past 6 months were reported by almost two-thirds of participants (510/821; 62.1%), with SARS-CoV-2 infection specifically associated with self-reported difficulty breathing, dizziness, fever, loss of smell, myalgia, rhinorrhea, and odynophagia; approximately half of the cases were completely asymptomatic. HIV status was not associated with differences in SARS-CoV-2 seroincidence or symptoms, and no behavioural or sociodemographic associations of infection were apparent. Pre-pandemic serology demonstrated antibodies recognizing one of SARS-CoV-2 Spike, RBD, or N in 151/994 participants (15.2%), but these were not associated with protection against subsequent SARS-CoV-2 infection. Overall, SARS-CoV-2 seroincidence was high early in the pandemic among Nairobi-based FSWs, with no clear socio-behavioural associations of infection.

## Introduction

In late 2019, there were reports of an atypical, pneumonia-like illness beginning to emerge in China, setting the stage for a global health crisis. By January 20^th^, 2020, the first confirmed case of SARS-CoV-2 infection was reported in the United States, followed by the first reports in Europe just days later, and Africa confirmed its first case on February 14^th^, 2020. However, the relative burden of COVID-19 disease and mortality during the first wave of the pandemic was low in sub-Saharan Africa (SSA) in comparison to Europe and North America. By 1^st^ February 2021, Europe reported 8.9 times more cases and 8.3 times more deaths than SSA; similarly, North America reported 8.8 and 7.5 times more cases and deaths than SSA, respectively [1]. This reduced burden of SARS-CoV-2 infection and mortality may relate in part to reduced public health surveillance and barriers to nasopharyngeal PCR testing in the region [2,3] since modelling suggests that by the end of 2021 only 1.4% of incident SARS-CoV-2 infections in SSA had been reported [4]. Indeed, retrospective sero-surveys and meta-analyses show that SARS-CoV-2 seroprevalence in African countries was around 40% [5] by October 2020 and upwards of 60% by September 2021 [6]. This indicates that transmission was more widespread than had been appreciated from case-based surveillance, but also suggests that a relatively high proportion of infections in SSA had been asymptomatic – a hypothesis that was subsequently confirmed [6,7].

In Kenya, the seroprevalence of SARS-CoV-2 increased from 4.3% to 48.5% between April 2020 and March 2021 [5,8–12], with approximately two-thirds of these cases being asymptomatic [13,14]. These sero-surveillance studies were conducted in different populations that included truck drivers, urban temporary settlement residents, antenatal care recipients and blood donors [5,9,10,15]. These studies were focused on reporting cross-sectional seroprevalence of SARS-CoV-2, rather than seroincidence, and provided little or no information regarding socio-behavioural associations of infection.

There are relatively few available data regarding the early spread of SARS-CoV-2 infection within female sex worker (FSW) populations, within Kenya or globally. There are an estimated 25 million [16] female sex workers (FSWs) in SSA [17], including ~170,000 residing in Kenya [18] and approximately 39,000 working in Nairobi [19]. FSWs are at high risk for sexually transmitted infections [20–22], but high levels of exposure to SARS-CoV-2 might also be expected due to intimate contact with multiple partners, solicitation of clients in crowded venues and difficulty negotiating mask use with clients [23]. If this is the case, then prevention efforts targeting FSW populations early in respiratory pandemics might have major public health benefits. While reports have shown that the COVID-19 pandemic had unintended social and economic consequences among FSWs, with health impacts such as unplanned pregnancies [23–26], there have been few studies specifically reporting the early seroincidence of SARS-CoV-2 among this population. Monitoring COVID-19 incidence in FSW populations using active case detection is difficult due to social and economic barriers to access healthcare services [27–29] and possible low uptake of COVID-19 testing due to the economic impact of state-mandated quarantine, although one sero-survey focused on FSW populations in Denmark [30] demonstrated a high seroprevalence in those who reported engaging in sex work.

In this study, we describe the seroincidence, socio-behavioural associations and clinical symptoms of SARS-CoV-2 infection in FSWs from Nairobi, Kenya early in the pandemic, between June 2020 – February 2021.

## Methods

### Ethics approval

The research was approved by the University of Toronto Health Sciences Research Ethics Board (RIS Approval #41580) and the Kenyatta National Hospital – University of Nairobi Ethics and Research Committee (KNH ERC #P514/06/2024).

### Study design and sampling

Participating FSWs were randomly recruited from June 14^th^ 2019 – November 26^th^ 2019 from seven Sex Worker Outreach Program (SWOP) clinics across Nairobi, Kenya, with numbers enrolled proportional to clinic size [31]. Participants were voluntarily enrolled and provided written consent. Eligibility criteria included attendance at one of the SWOP clinics in the past 12 months prior to the start of the study, being active in sex work, being between 18–45 years old, not pregnant or breastfeeding, and not having a chronic illness (other than HIV) that was likely to alter host immunology. Women were recruited through peer educators and community mobilizers who were based at the SWOP clinics. Baseline behavioural-biological surveys took place from June – December 2019, prior to the global SARS-CoV-2 pandemic, with follow-up behavioural-biological surveys conducted June 2020 – February 2021, 3–11 months after the first confirmed COVID-19 case in Kenya. Whole blood was collected at both visits, which were subsequently separated into plasma and peripheral mononuclear blood cells (PBMCs) and cryo-preserved for future use. Cryo-preserved biological samples were accessed between April 2022 – June 2022 for serological testing. Women testing positive for HIV were provided with counselling and access to HIV care. The behavioural questionnaires captured data on sex work activities and behaviours, age, living conditions, region of residence, partner histories, alcohol and other drugs use, food security, as well as recent exposure to violence, mental health morbidities and reproductive health. At the follow-up survey, questions were included regarding participant experience of 17 respiratory and gastrointestinal symptoms since 1^st^ January 2020 (diarrhea, difficulty breathing, odynophagia, dizziness, exhaustion, eye pain, fever, headache, loss of appetite, loss of smell, myalgia, nausea, new cough, rhinorrhea, shivering, stomach pain, sore throat). Questions regarding COVID-19 testing, and government mandated isolation for COVID-19 were also included. All questionnaire and clinical data were stored on password-protected secure servers.

### Diagnostic tests and sampling

HIV testing was performed for all participants by rapid HIV test, with positive tests confirmed using HIV DNA Genexpert. Whole blood was collected at each visit and centrifuged at room temperature for 10 minutes x 1600 rpm; the plasma layer was separated and aliquoted into 2x 1mL cryovials and stored at -80C. Density gradient separation (Ficoll Paque) was used to isolate PBMCs which were cryopreserved at -150C in FBS + 10% DMSO. Plasma and PBMCs were then shipped to Toronto, Canada for immunological assays.

### SARS-CoV-2 serology

During the research planning phase significant community concerns were voiced regarding any research that involved real-time testing for SARS-CoV-2 infection by PCR, due to the financial implications of government-mandated quarantine; in addition, limited resources were available for frequent, large-scale PCR testing of asymptomatic participants. Therefore, we opted to complete serological testing retrospectively, after study completion. SARS-CoV-2 serology was performed using cryopreserved plasma, as previously described [32]. Briefly, chemiluminescent ELISA was used to detect total IgG antibody levels to full-length spike trimer, its receptor binding domain (RBD) and nucleocapsid (N). LUMITRAC 600 high-binding white polystyrene 384-well microplates (Greiner Bio-One, #781074; VWR, #82051–268) were pre-coated overnight with 10 µL per well of antigen (Ag): 50 ng spike (SmT1), 20 ng RBD (331–521) and 7 ng N, all supplied by the National Research Council of Canada (NRC). The next day, the assay was performed at room temperature with washing four times in 100 µL PBS-T before each of the following four steps: [1] wells were blocked for 1 h in 80 µL 5% Blocker BLOTTO (ThermoFisher Scientific, #37530); [2] 10 µL of serum diluted 1:160 or 1:2,560 in 1% final Blocker BLOTTO in PBS-T was added and incubated for 2 h; [3] 10 µL of a human anti-IgG fused to HRP (IgG#5 by NRC, 0.9 ng/well) diluted in 1% final Blocker BLOTTO in PBS-T was added, followed by a 1-h incubation; [4] 10 µL of ELISA Pico Chemiluminescent Substrate (ThermoFisher Scientific, #37069, diluted 1:4 in MilliQ distilled H_2_O) was added and incubated for 5–8 min. Chemiluminescence was read on an EnVision 2105 Multimode Plate Reader (Perkin Elmer) plate reader at 100 ms/well using an ultra-sensitive detector. Raw chemiluminescent values were normalized to a synthetic standard included on each assay plate (VHH72-Fc supplied by NRC for spike/RBD or an anti-nucleocapsid IgG Ab from Genscript, #A02039), and these relative ratios were further converted to binding Ab units (BAU/mL) using the WHO International Standard 20/136 as the calibrant [32]. Positivity thresholds were determined for the 1:160 dilution using 3 SD from the mean of control samples as previously described [30]. Seroincident infection was defined the presence of antibodies against at least 2 out of 3 tested antigens at follow-up, among FSWs who had been seronegative at baseline.

### Statistical analysis

Self-reported symptoms and socio-demographic variables, including HIV status, were assessed for associations with SARS-CoV-2 infection. Chi-square tests were used to analyse categorical variables. Welch’s t-tests were used to analyse continuous variables. Mann-Whitney U tests were used to analyse ordinal variables. Associations between age and number of respiratory symptoms was analyzed using Pearson’s product-moment correlation test. Categorical variables were reported as counts with percentages. Continuous variables were reported as mean or median with the range. Dichotomous variables were reported as counts with percentages. All analyses were exploratory in nature, and so all p-values are unadjusted unless stated otherwise. Socio-demographic variables were considered for inclusion in multiple logistic regression analysis if they attained a significance <5%. As time between the start of the pandemic and follow-up visit was variable of interest, we did not adjust analyses for temporal variations. Two-sided p < 0.05 was considered statistically significant. Statistical analyses were conducted in R (Version 4.2.1 (2022-06-23) -- “Funny-Looking Kid”). Data storage was in Microsoft Excel for Mac (Version 16.70 (23021201).

## Results

### Participant demographics

**Fig 1:**
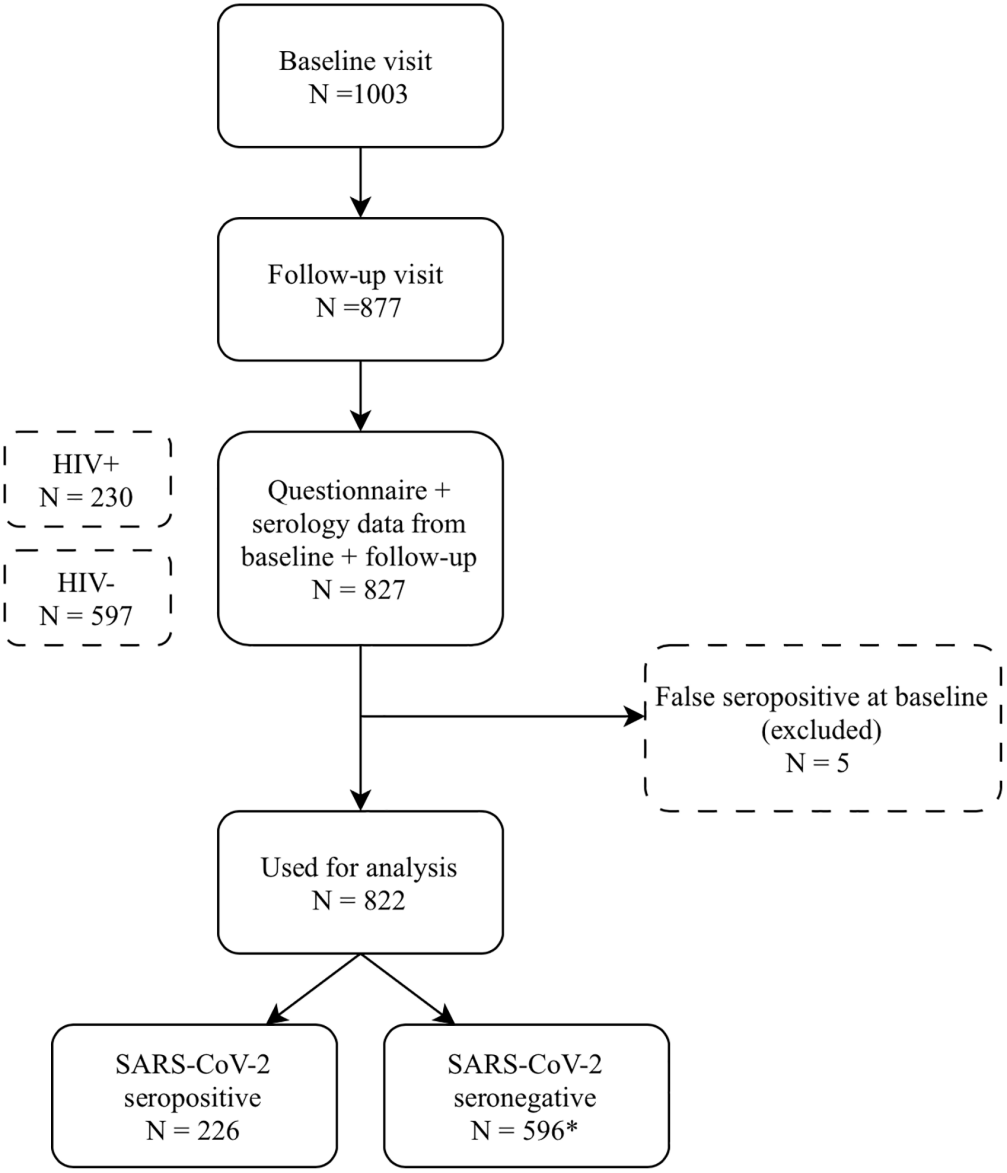
Study flow chart for study inclusion. There were 1003 participants enrolled, 827 with serology at baseline and follow-up. *Of the 596 seronegative participants, 595 provided symptomatology data.

Of the 1003 participants recruited into the original protocol, 877 participants had at least one follow-up visit after the declaration of the SARS-CoV-2 pandemic in March 2020; there were biological samples available for SARS-CoV-2 serology in 827, and so this subset constituted our primary analytic cohort (Fig 1). The mean participant age was 32.7 years (range; 18–45 years). HIV prevalence at baseline was 27.8% (230/827), and 98.3% of participants living with HIV were accessing antiretroviral treatment. At the time of the follow-up visit when SARS-CoV-2 serology was performed, the average number of clients women had seen over the past 7 days was 2.97 (0–60), and nearly half (49.6%) reported that their main place for sex work solicitation was the phone/mobile or over the internet. The great majority of participants reported stable housing (95.3%), which was shared in 85.7% with an average of 2.6 people per shared household. Recent substance use (past 3 months) was reported by 63% of participants for alcohol, amphetamine use by 29.3% and cannabis use by 18.2%. Use of cocaine, hallucinogens, opioids, injectables, or sedatives was asked but relatively few people reported using any therefore these were not assessed further (Table 1). At follow-up, 142 of the 877 (16.2%) participants who completed a questionnaire reported ever having accessed SARS-CoV-2 testing services at government-approved facilities; of those only 5 (0.6%) had tested positive, and 4 (0.4%) reported a period of state-mandated quarantine. No participant had been vaccinated against SARS-CoV-2 at the time of study follow-up.

**Table 1:** Demographics and behavioural characteristics.

Demographic	Participants (n = 827)
Age (mean, range)	32.7 (18-45)
Living with HIV Suppressed viral load*	230 (27.8%) 177 (77.0%)
ART usage	226 (98.3%)
*Sex work*	
Number of clients, past 7 days (mean, range)	2.97 (0-60)
Main place to solicit sex work, past 3 months Phone/mobile/internet Sex den/brothel/escort service/massage parlour Bar/club/restaurant/lodge/hotel/social gathering Street/bus/taxi/truck stand Home/middleman/markets	410 (49.6%) 16 (1.9%) 163 (19.7%) 117 (14.1%) 17 (2.0%)
*Living*	
Visit outside of Nairobi, past 6 months	285 (34.5%)
Moved house, past 3 months	236 (28.5%)
Regular place to stay	788 (95.3%)
Living with others	709 (85.7%)
Number of people in home (mean)	2.6
Number of sleeping rooms in home (mean)	1.3
*Substance use*	
Any alcohol use, last 3 months	521 (63.0%)
Any cannabis use, last 3 months	151 (18.2%)
Any amphetamine use, last 3 months	242 (29.3%)

*Suppressed viral load: viral load less than 200 copies/ml

### SARS-CoV-2 seroconversion during the study period

SARS-CoV-2 serology was completed for all participants with plasma samples available at baseline (n = 994) and study completion (n = 827, 83.2%). Positive SARS-CoV-2 serology was defined as the detection of antibodies recognizing at least 2 out of the 3 antigens tested, as previously described [32], and negative serology was defined as the detection of antibodies recognizing ≤1 of the 3 antigens tested. At baseline (pre-pandemic) 6/994 (0.6%) participants were SARS-CoV-2 seropositive (≥2 antigens); these were assumed to be biologic false positives as these samples were collected before the start of the COVID-19 pandemic. Five of these participants returned for a follow-up visit and were excluded from subsequent analysis (Fig 1)(Table 2). Among all participants at baseline, 145/994 (14.5%) recognized only 1 of the 3 antigens, and 843/994 (84.8%) had no response to any of the 3 antigens.

**Table 2:** SARS-CoV-2 sero-status at baseline and follow-up visits.

	Baseline (n = 994)	Follow-up (n = 827)
**Seropositive**	**6 (0.6%)**	**229 (27.7%)**
All 3 antigens Spike/RBD Spike/N RBD/N	0 0 6 (0.6%) 0	140 (16.9%) 31 (3.7%) 57 (6.9%) 1 (0.1%)
**Seronegative**	**988 (99.4%)**	**598 (72.3%)**
No antigens Spike only RBD only N only	843 (84.8%) 61 (6.1%) 2 (0.2%) 82 (8.2%)	469 (56.7%) 87 (10.5%) 1 (0.1%) 41 (5.0%)

SARS-CoV-2 seroprevalence was 27.7% (229/827 participants) at the time of follow-up. Antibody recognition of the 3 viral antigens was as follows: Spike/RBD/N (140, 16.9%), Spike/RBD (31, 3.7%), Spike/N (57, 6.9%), and RBD/N (1, 0.1%) (Table 2). Follow up serology was performed a median of 201 days after 11-Mar-2020, the date when COVID-19 was officially declared a pandemic in Kenya: as expected, SARS-CoV-2 seropositivity was strongly associated with the number of days after this date that the follow-up sample was collected (p < 0.0001; Table 3).

**Table 3:** COVID-19 symptoms & associations with seroconversion.

COVID-19 symptoms & time of acquisition	SARS-CoV-2 infection (n = 226)	No infection (n = 595)	p-value (unadjusted)	OR
Any symptom	154 (68.14%)	356 (59.83%)	0.035	1.43
Total number of symptoms (mean)	2.55	1.84	0.003	
Diarrhea	21 (9.29%)	35 (5.88%)	0.115	
Difficulty breathing	26 (11.50%)	38 (6.39%)	0.022	1.90
Odynophagia	18 (7.96%)	28 (4.70%)	0.100	
Dizziness	28 (12.39%)	45 (7.56%)	0.042	1.73
Exhaustion	46 (20.35%)	87 (14.62%)	0.059	
Eye pain	3 (1.33%)	4 (0.67%)	0.625	
Fever	45 (19.91%)	68 (11.43%)	0.002	1.92
Headache	91 (40.26%)	199 (33.50%)	0.084	
Loss of appetite	34 (15.04%)	68 (11.43%)	0.199	
Loss of smell	18 (8.00%)	21 (3.53%)	0.012	2.37
Myalgia	40 (17.70%)	61 (10.25%)	0.005	1.88
Nausea	15 (6.64%)	30 (5.04%)	0.468	
New cough	52 (23.00%)	128 (21.51%)	0.713	
Rhinorrhea	62 (27.43%)	119 (20.00%)	0.028	1.51
Shivering	31 (13.71%)	54 (9.08%)	0.068	
Stomach pain	29 (12.83%)	78 (13.11%)	1	
Sore throat	35 (15.49%)	52 (8.74%)	0.007	1.91
Timing				
Days between start of pandemic and visit date (median, range)	217 (97-342)	190 (92-324)	<0.0001	

Symptom data were available for 595/596 seronegative participants (821/822 of all participants).

Start of pandemic March 11^th^, 2020

The proportion of participants who were SARS-CoV-2 seropositive increased steadily over time, as expected. During the first period after reopening of the clinic, which happened 91 days after declaration of the pandemic in Kenya, 32/211 participants (15.2%) tested were seropositive; this increased to 127/365 seropositive (34.8%) of participants tested later in the pandemic (Table 4).

**Table 4:** Rates of seroincidence during follow-up period.

Days since start of pandemic	# of seroconverters/total participants at time period
Pre-pandemic	0.6% (6/994)
0-91 days	No data (clinic closed)
92-134 days	15.2% (32/211)
135-169 days	25.7% (28/109)
170-205 days	27.5% (39/142)
>205 days	34.8% (127/365)

Start of the pandemic defined as March 11^th^, 2020

Percentages represent the number participants who tested seropositive for SARS-CoV-2 compared to total number of participants that attended the clinic at the specified time intervals.

**Fig 2:**
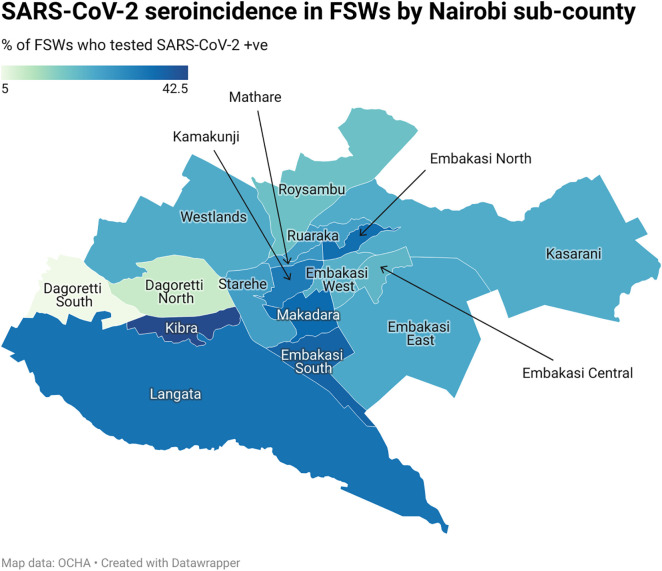
SARS-CoV-2 seroincidence in FSWs by Nairobi sub-county. Seroincidence rates based on individuals who tested SARS-CoV-2 seropositive at their final visit as a percentage of all participants who attended the final visit from all 17 sub-counties.

SARS-CoV-2 incidence among participants was similar (~25%) across the 17 Nairobi sub-counties, although two sub-counties, Dagoretti South and Dagoretti North, demonstrated lower seroincidence rates of 5% and 9.38%, respectively (Fig 2). Pre-existing antibodies recognizing only one of SARS-CoV-2 Spike, RBD, and N were not associated with protection against subsequent SARS-CoV-2 infections. There was no evidence of altered SARS-CoV-2 seroincidence among the 15.3% (126/822) of follow-up participants who had antibodies against 1 of the 3 antigens at their pre-pandemic baseline visit. Specifically, among these participants, the proportion of participants who remained seronegative (n = 91/596, 15.27%) was similar to those who seroconverted (n = 35/226, 15.49%) at study exit (Table 5).

**Table 5:** Socio-behavioural & baseline serological associations of infection.

Total n = 822	SARS-CoV-2 infection (n = 226)	No infection (n = 596)	p-value (unadjusted)
Age (mean)	32.32	32.89	0.339
Living with HIV	56 (24.8%)	171 (28.7%)	0.302
*Sex work*			
Number of clients, past 7 days (mean)	3.19	2.89	0.383
Main place to solicit sex work, past 3 months Phone/mobile/internet Sex den/brothel/escort service/massage parlour Bar/club/restaurant/lodge/hotel/social gathering Street/bus/taxi/truck stand Home/middleman/markets	115 (50.9%) 6 (2.6%) 51 (22.6%) 30 (13.3%) 2 (0.9%)	291 (48.8%) 10 (4.4%) 111 (49.1%) 87 (38.5%) 15 (6.6%)	0.348
*Living*			
Visit outside of Nairobi, past 6 months	70 (31.0%)	215 (36.1%)	0.186
Moved house, past 3 months	74 (32.7%)	160 (26.8%)	0.116
Regular place to stay	215 (95.1%)	568 (95.3%)	0.988
Living with others	194 (85.8%)	511 (85.7%)	1
Number of people in home (mean)	2.68	2.59	0.569
Number of sleeping rooms in home (mean)	1.20	1.32	0.021
*Substance use*			
Any alcohol use, last 3 months	145 (64.2%)	376 (63.1%)	0.776
Any cannabis use, last 3 months	37 (16.4%)	114 (19.1%)	0.350
Any amphetamine use, last 3 months	73 (32.3%)	169 (28.4%)	0.277
*Antibodies at baseline*			
Baseline antibodies vs. any of the 3 antigens Spike alone RBD alone N alone	35 (15.49%) 12 (5.31%) 1 (0.44%) 22 (9.73%)	91 (15.27%) 43 (7.21%) 1 (0.17%) 47 (7.88%)	1

### Symptoms attributable to SARS-CoV-2 infection

A follow-up questionnaire administered at the same time as follow-up blood sampling asked about participant experience of 17 respiratory and gastrointestinal symptoms since 1^st^ January 2020. These symptoms were common across the cohort regardless of SARS-CoV-2 serostatus at follow-up; this may have been due to symptoms occurring due to infection by other common upper respiratory viruses (influenza, RSV, rhinovirus, etc.) during the follow-up period. Overall, 510 (62.12%) of all cohort participants experienced at least 1 symptom during this period, with headache (35.32%), new cough (21.92%), and rhinorrhea (22.05%) being the most common. However, the proportion of individuals reporting any symptoms was higher among SARS-CoV-2 seroconverters compared with those who did not acquire infection (154/226, 68.14% vs. 356/596, 59.73%; p = 0.035) (Table 3). In addition, the total number of symptoms experienced was greater among seroconverters in comparison to those who remained SARS-CoV-2 uninfected (2.55 vs. 1.84 symptoms; p = 0.003). Seven of the 17 symptoms were specifically enriched among SARS-CoV-2 seroconverters, specifically: difficulty breathing (26/226 vs. 38/595; p = 0.022), dizziness (28/226 vs. 45/595; p = 0.042), fever (45/226 vs. 45/595; p = 0.002), loss of smell (18/226 vs. 21/595; p = 0.012), muscle pain (40/226 vs. 61/595; p = 0.005), coryza (62/226 vs. 119/595; p = 0.028), and sore throat (35/226 vs. 52/595; p = 0.007) (Table 3). Subsequently, symptomatic disease for the purpose of this analysis was defined as having at least one of these seven significantly associated symptoms (i.e.,: difficulty breathing, dizziness, fever, loss of smell, muscle pain, running nose, or sore throat).

### Socio-demographic associations of SARS-CoV-2 seroconversion

We next examined if follow-up socio-demographic variables were associated with SARS-CoV-2 seroconversion. As detailed in Table 4, we did not find associations between SARS-CoV-2 seroincidence and age, HIV status or the average number of clients seen in the past 7 days, household parameters, travel outside of Nairobi within the last 6 months, or recent substance use within the last 3 months (Table 5). Having a reduced number of sleeping room in the home was associated with seroconversion (1.20 rooms vs. 1.32 rooms; p = 0.021), however this association did not remain significant after correcting for multiple comparisons (p > 0.05). Socio-behavioural variables were selected from the follow-up survey. Overall, no clear socio-behavioural associations of SARS-CoV-2 infection were identified among this cohort of women who sell sex in Nairobi.

### Associations of symptomatic infection among seroconverters

**Fig 3:**
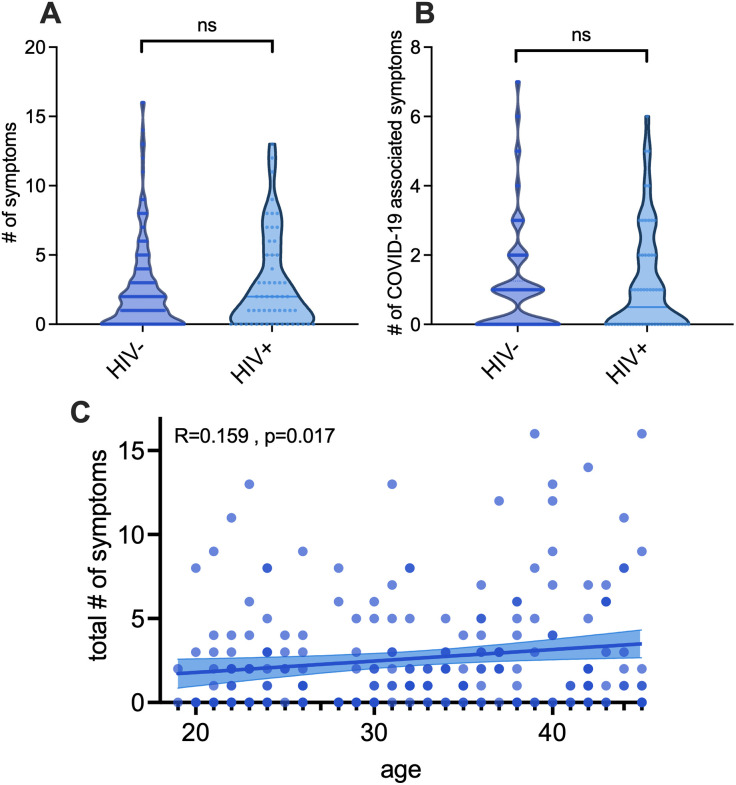
Biological associations of symptoms in SARS-CoV-2 seroconverters. A. Associations of total symptoms with HIV status in SARS-CoV-2 seroconverters. B. Associations of SARS-CoV-2 specific symptoms with HIV status in SARS-CoV-2 seroconverters. C. Correlation between age and number of SARS-CoV-2 symptoms.

We next assessed associations with symptomatic disease among the SARS-CoV-2 seroconverters (n = 226). HIV status was not associated with the number of self-reported symptoms, either with the total repertoire of symptoms (Fig 3A) or with the seven SARS-CoV-2 specific symptoms (Fig 3B); neither was HIV status associated with the presence of at least one SARS-CoV-2 specific symptom (i.e.,: with symptomatic infection; Table 6). However, increasing age was significantly correlated, albeit weakly, with an increased total number of symptoms (R = 0.1587, p = 0.017, Fig 3C).

**Table 6:** Socio-behavioural associations of COVID-19 symptoms among SARS-CoV-2 seroconverters.

	Symptomatic (n = 119)	Asymptomatic (n = 107)	p-value (unadjusted)	OR
HIV+	28 (23.5%)	28 (26.2%)	0.761	
*Sex work*				
Number of clients, past 7 days (mean)	2.92	3.49	0.321	
Main place to solicit sex work, past 3 months Phone/mobile/internet Sex den/brothel/escort service/massage parlour/ bar/club/restaurant/lodge/hotel/social gathering street/bus/taxi/truck stand home/middleman/markets	68 (57.1%) 4 (3.4%) 24 (20.2%) 13 (10.9%) 2 (1.7%)	47 (43.9%) 2 (1.9%) 27 (25.2%) 17 (15.9%) 0 (0%)	0.144	
*Living*				
Visit outside of Nairobi, past 6 months	41 (34.4%)	28 (26.2%)	0.294	
Moved house, past 3 months	48 (40.3%)	26 (24.3%)	0.015	2.09
Regular place to stay	113 (95.0%)	102 (95.3%)	1	
Living with others	99 (83.2%)	95 (88.8%)	0.311	
Number of people in home (mean)	2.81	2.54	0.277	
Number of sleeping rooms in home (mean)	1.19	1.22	0.685	

No specific sex-work related factors, including client number, were associated with symptomatic disease among participants with SARS-CoV-2 infection (Table 6), and likewise there were no clear socio-behavioural associations. Although moving homes within the past 3 months was associated with symptomatic disease (40.3%, 48/119 of symptomatic seroconverters vs. 24.3%, 26/107 of asymptomatic; p = 0.015, OR=2.09), this association did not remain significant after correction for multiple comparisons (p > 0.05).

## Discussion

Our understanding of COVID-19 transmission in SSA during the early stages of the pandemic remains limited, due in part to underreporting and to reduced public health and testing infrastructure. Sero-surveys in Kenya were limited in breadth of coverage and did not include key populations such as female sex workers. This study helps to bridge these knowledge gaps by focusing on the burden of COVID-19 in FSWs in Nairobi, Kenya. We show that the seroincidence within FSWs in Kenya was high and similar to previously reported numbers in the general population, with a substantial portion of infections being asymptomatic. Unexpectedly, we could not identify clear socio-behavioural risk factors of infection, with no links between parameters such as client numbers or place of work and seroincidence.

SARS-CoV-2 infection was common in this community of Nairobi FSWs early in the COVID-19 pandemic, with the 27.7% seroprevalence by Feb-2021 being high in comparison to the 4.3% to 9.1% seen in blood donors [8,10] between June – September 2020. This rate, however, is similar to the ~ 40% seroprevalence seen in some other key populations between October – December 2020, such as truck drivers and individuals living in informal settlements where face-to-face contact is high and protective behaviours may not be possible [5,9]. Seropositivity rose steadily over time in our cohort; among participants sampled after September 2020, 34.8% attending the clinic were seropositive (Table 4), compared to 15.2% of participants who were sampled earlier, between June to July 2020. The high seroincidence seen in FSWs early in the pandemic highlights the importance of designating sex workers as a priority population for future sero-surveillance.

In our study a considerable fraction of cases were asymptomatic: 31.86% of cases reported no preceding symptoms at all, and 47.8% reported that they had not experienced any of the 7 symptoms significantly associated with SARS-CoV-2 seroconversion. Importantly, rates of asymptomatic infection may actually have been much higher than this: because the precise timing of SARS-CoV-2 infection was not known, participants were asked to recall any respiratory symptoms since Jan 2020, and these symptoms could have been due to respiratory infections other than SARS-CoV-2. In keeping with this, “any symptoms” were reported during this period by over half (59.7%) of participants who had remained SARS-CoV-2 seronegative. Reporting bias may also contribute to inaccurate recount of symptomatology as the stigma and fear surrounding COVID-19 infections, and the threat of mandated quarantine in poorly maintained government facilities was highly prevalent. Symptoms among seroconverters were generally mild, with most seropositive participants reporting less than 3 symptoms, but due to the lack of SARS-CoV-2 qPCR testing we were unable to link symptoms to a specific infection episode. Our behavioural survey did not include questions regarding severe COVID-19 disease outcomes such as hospital or ICU admissions, but we did not receive any personal communications regarding hospital visits or severe outcomes due to COVID-19 through our peer outreach network.

We had originally hypothesized that FSWs would be at high risk for COVID-19 due to mixing patterns associated with sex work; while seroincidence was high, the lack of clear associations with socio-behavioural factors was unexpected, particularly given previously reported behavioural associations [33–38]. However, there may be numerous other social situations that may expose participants to respiratory droplets that are unrelated to sex work and that were not captured in the questionnaire, beyond the physical act of sex work. We did not find that age was associated with SARS-CoV-2 incidence, but this may be explained in part by the limited age range (18-45 years), with lack of individuals who would be considered high-risk for severe disease (i.e.,: >60 years). This consideration is particularly relevant in understanding the dynamics of COVID-19 spread in key populations such as FSWs, where older adults may be underrepresented. HIV status was also not associated with SARS-CoV-2 incidence, although few participants were significantly immunosuppressed due to very high uptake of antiretroviral therapy. Although the association of a lower number of sleeping rooms in the home with SARS-CoV-2 seroconversion was in keeping with prior findings that a shared bedroom is associated with SARS-CoV-2 transmission [36], this did not remain significant when correcting for multiple comparisons (False Discovery Rate; adjusted p >0.05). While a previous study found that food insecurity was linked to SARS-CoV-2 seroconversion among FSWs [37], we were unable to confirm this despite collecting extensive data in this regard. Overall, the lack of associations between SARS-CoV-2 infection and the socio-behavioral factors that we assessed suggests that additional factors not specific to sex work are likely to be important determinants of infection, since our questionnaire was quite focused on parameters of sex work and intimate partner violence.

Our definition of SARS-CoV-2 seropositivity required the presence of antibodies recognizing at least 2 out of 3 viral antigens, as previously described [32]. While the very low rate of false-positive serology in pre-pandemic samples (6/994; 0.6%) validates this approach, antibodies recognizing a single viral antigen were detected at baseline in 145/994 participants (14.6%). This is most likely due to cross-reactivity from previous exposures to other seasonal coronaviruses, of which four are in global circulation [39]. Among the three tested antigens, N exhibited the highest cross-reactivity (88/994; 8.8%) while RBD had the lowest (2/994; 0.2%), in keeping with the fact that the RBD is not highly conserved across the coronavirus family [40]. Interestingly, the pre-pandemic detection of antibodies recognizing a single viral antigen was not associated with subsequent protection against SARS-CoV-2 infection (Table 5).

Our study does have several limitations. SARS-CoV-2 infection was determined by serology, and so we may have missed some infections due to waning of antibody titres [41]. Additionally, the timing of participant follow-up varied widely, from just three months after declaration of the pandemic to more than seven months, and so many participants who were screened early will subsequently have acquired infection. Later follow up may also have affected the ability of participants to recall prior respiratory symptoms. Since this cohort was entirely composed of female participants from a specific profession, our findings cannot be generalized to sub-Saharan Africa as a whole. Due to the observational nature of this study and the fact that our questionnaire included limited questions beyond sex work and intimate partner violence, we may have missed important socio-behavioural associations of SARS-CoV-2 infection. Finally, we acknowledge that a larger sample size might have enabled the detection of more moderate, but still valid, associations of infection.

This study provides important information regarding the burden of COVID-19 in a high-risk population of FSWs in Nairobi, with evidence of considerable transmission in the early days of the pandemic. Although our study was not ideally designed to assess the proportion of asymptomatic infection, symptoms tended to be mild, and infection was not linked to sex work related factors such as client numbers or work venue. FSWs, while comprising a small portion of the total population of Kenya, may represent an important subgroup for disease surveillance due to high rates of social contact. Understanding the epidemiology of emerging infectious diseases in these and other key populations where social distancing (and other infection prevention strategies) is not feasible may be crucial for effective pandemic planning and vaccine deployment. Our goal is to inform public health and pandemic preparedness policies that address the unique needs for the health and safety of FSWs, and our study demonstrates that FSW communities can be included in such work without alienating this marginalized group. FSWs face unique challenges in the face of pandemics, including the criminalization of their profession, financial instability in times of mandatory reduced social contact, and an inability to participate in social distancing, but this need not lead to their exclusion.

## Supporting information

S1 File Questionnaire on inclusivity in global research. (DOCX)

## References

[1] Our World in Data. Cumulative confirmed COVID-19 cases by world region. Accessed 2023 June 18. https://ourworldindata.org/grapher/cumulative-covid-cases-region

[2] Adebisi YA, Rabe A, Lucero-Prisno Iii DE. COVID-19 surveillance systems in African countries. Health Promot Perspect. 2021;11(4):382–92. doi: 10.34172/hpp.2021.49 35079582 PMC8767077

[3] Tessema GA, Kinfu Y, Dachew BA, Tesema AG, Assefa Y, Alene KA, et al. The COVID-19 pandemic and healthcare systems in Africa: a scoping review of preparedness, impact and response. BMJ Glob Health. 2021;6(12):e007179. doi: 10.1136/bmjgh-2021-007179 34853031 PMC8637314

[4] Cabore JW, Karamagi HC, Kipruto HK, Mungatu JK, Asamani JA, Droti B, et al. COVID-19 in the 47 countries of the WHO African region: a modelling analysis of past trends and future patterns. Lancet Glob Health. 2022;10(8):e1099–114. doi: 10.1016/S2214-109X(22)00233-9 35659911 PMC9159735

[5] Kagucia EW, Gitonga JN, Kalu C, Ochomo E, Ochieng B, Kuya N. Anti-severe acute respiratory syndrome coronavirus 2 immunoglobulin G antibody seroprevalence among truck drivers and assistants in Kenya. Open Forum Infectious Diseases. 2021;8(7):ofab314. doi: 10.1093/ofid/ofab314 PMC8519263 34660838

[6] Lewis HC, Ware H, Whelan M, Subissi L, Li Z, Ma X, et al. SARS-CoV-2 infection in Africa: a systematic review and meta-analysis of standardised seroprevalence studies, from January 2020 to December 2021. BMJ Global Health. 2022;7(8). 10.1136/bmjgh-2022-008793 PMC9402450 35998978

[7] Abraha HE, Gessesse Z, Gebrecherkos T, Kebede Y, Weldegiargis AW, Tequare MH, et al. Clinical features and risk factors associated with morbidity and mortality among patients with COVID-19 in northern Ethiopia. Int J Infect Dis. 2021;105:776–83. doi: 10.1016/j.ijid.2021.03.037 33741488 PMC7962557

[8] Adetifa IMO, Uyoga S, Gitonga JN, Mugo D, Otiende M, Nyagwange J, et al. Temporal trends of SARS-CoV-2 seroprevalence during the first wave of the COVID-19 epidemic in Kenya. Nat Commun. 2021;12(1):3966. doi: 10.1038/s41467-021-24062-3 34172732 PMC8233334

[9] Munywoki PK, Nasimiyu C, Alando MD, Otieno N, Ombok C, Njoroge R, et al. Seroprevalence and risk factors of SARS-CoV-2 infection in an urban informal settlement in Nairobi, Kenya, December 2020. F1000Res. 2021;10:853. doi: 10.12688/f1000research.72914.2 35528961 PMC9065925

[10] Uyoga S, Adetifa IMO, Karanja HK, Nyagwange J, Tuju J, Wanjiku P, et al. Seroprevalence of anti-SARS-CoV-2 IgG antibodies in Kenyan blood donors. Science. 2021;371(6524):79–82. doi: 10.1126/science.abe1916 33177105 PMC7877494

[11] Etyang AO, Adetifa I, Omore R, Misore T, Ziraba AK, Ng’oda MA, et al. SARS-CoV-2 seroprevalence in three Kenyan health and demographic surveillance sites, December 2020-May 2021. PLOS Glob Public Health. 2022;2(8):e0000883. doi: 10.1371/journal.pgph.0000883 36962821 PMC10021917

[12] Uyoga S, Adetifa IMO, Otiende M, Yegon C, Agweyu A, Warimwe GM. Prevalence of SARS-CoV-2 antibodies from a national serosurveillance of Kenyan Blood Donors, January-March 2021. JAMA. 2021;326(14):1436–8. 34473191 10.1001/jama.2021.15265 PMC8414357

[13] Ngere P, Onsongo J, Langat D, Nzioka E, Mudachi F, Kadivane S, et al. Characterization of COVID-19 cases in the early phase (March to July 2020) of the pandemic in Kenya. J Glob Health. 2022;12:15001. doi: 10.7189/jogh.12.15001 36583253 PMC9801068

[14] Ministry of Health. Covid-19. Accessed 2024 January 17. https://www.health.go.ke/covid-19

[15] Sero-surveillance for IgG to SARS-CoV-2 at antenatal care clinics in two Kenyan referral hospitals. medRxiv. https://www.medrxiv.org/content/10.1101/2021.02.05.21250735v1.full 10.1371/journal.pone.0265478 PMC9565697 36240176

[16] Key population mapping and size estimation in selected counties in Kenya: phase 1: key findings. https://stacks.cdc.gov/view/cdc/164651

[17] Laga I, Niu X, Rucinski K, Baral S, Rao A, Chen D, et al. Mapping the number of female sex workers in countries across sub-Saharan Africa. Proc Natl Acad Sci U S A. 2023;120(2):e2200633120. doi: 10.1073/pnas.2200633120 36595685 PMC9926247

[18] UNFPA ESARO. Report on the female sex workers program in Kenya 2018 to 2021. 2022. https://esaro.unfpa.org/en/publications/report-female-sex-workers-program-kenya-2018-2021

[19] Enumeration of sex workers in the central business district of Nairobi, Kenya | PLOS ONE. https://journals.plos.org/plosone/article?id=10.1371/journal.pone.0054354 10.1371/journal.pone.0054354 PMC3556081 23372713

[20] Report on global sexually transmitted infection surveillance. 2018. https://www.who.int/publications-detail-redirect/9789241565691

[21] Seib C, Debattista J, Fischer J, Dunne M, Najman JM. Sexually transmissible infections among sex workers and their clients: variation in prevalence between sectors of the industry. Sex Health. 2009;6(1):45–50. doi: 10.1071/sh08038 19254491

[22] Cárcamo CP, Campos PE, García PJ, Hughes JP, Garnett GP, Holmes KK, et al. Prevalences of sexually transmitted infections in young adults and female sex workers in Peru: a national population-based survey. Lancet Infect Dis. 2012;12(10):765–73. doi: 10.1016/S1473-3099(12)70144-5 22878023 PMC3459082

[23] Machingura F, Chabata ST, Busza J, Jamali G, Makamba M, Dirawo J. Potential reduction in female sex workers’ risk of contracting HIV during coronavirus disease 2019. AIDS. 2021;35(11):1871. 33973873 10.1097/QAD.0000000000002943 PMC8373442

[24] Kavanagh NM, Marcus N, Bosire R, Otieno B, Bair EF, Agot K, et al. Health and economic outcomes associated with COVID-19 in women at high risk of HIV infection in rural Kenya. JAMA Netw Open. 2021;4(6):e2113787. doi: 10.1001/jamanetworkopen.2021.13787 34137826 PMC12578490

[25] Kimani J, Adhiambo J, Kasiba R, Mwangi P, Were V, Mathenge J, et al. The effects of COVID-19 on the health and socio-economic security of sex workers in Nairobi, Kenya: emerging intersections with HIV. Glob Public Health. 2020;15(7):1073–82. doi: 10.1080/17441692.2020.1770831 32459578

[26] Modisaotsile I, Stacey M, Odek W, Ogutu D, Kindyomunda R. Heightened risk of unintended pregnancy among sex workers and sex worker organizations’ response during the stringent COVID-19 containment measures in East and Southern Africa. China Popul Dev Stud. 2023;7(1):37–47. doi: 10.1007/s42379-023-00128-1 37193367 PMC10072027

[27] Potter LC, Horwood J, Feder G. Access to healthcare for street sex workers in the UK: perspectives and best practice guidance from a national cross-sectional survey of frontline workers. BMC Health Serv Res. 2022 Feb 11;22(1):178. 35148761 10.1186/s12913-022-07581-7 PMC8840502

[28] Kurtz SP, Surratt HL, Kiley MC, Inciardi JA. Barriers to health and social services for street-based sex workers. J Health Care Poor Underserved. 2005;16(2):345–61. doi: 10.1353/hpu.2005.0038 15937397

[29] UNFPA ESARO. A Rapid Scoping Assessment of the Impact of COVID-19 on Sex Worker Programmes in East and Southern Africa. 2021. Accessed 2024 January 17. https://esaro.unfpa.org/en/publications/rapid-scoping-assessment-impact-covid-19-sex-worker-programmes-east-and-southern-africa

[30] Eriksen ARR, Fogh K, Hasselbalch RB, Bundgaard H, Nielsen SD, Jørgensen CS, et al. SARS-CoV-2 antibody prevalence among homeless people and shelter workers in Denmark: a nationwide cross-sectional study. BMC Public Health. 2022;22(1):1261. doi: 10.1186/s12889-022-13642-7 35761270 PMC9238223

[31] Kung’u M, Kabuti R, Babu H, On Behalf Of The Maisha Fiti Study Champions, Nyamweya C, Okumu M, et al. Conducting violence and mental health research with female sex workers during the COVID-19 pandemic: ethical considerations, challenges, and lessons learned from the maisha fiti study in Nairobi, Kenya. Int J Environ Res Public Health. 2023;20(11):5925. doi: 10.3390/ijerph20115925 37297529 PMC10252611

[32] Colwill K, Galipeau Y, Stuible M, Gervais C, Arnold C, Rathod B. A scalable serology solution for profiling humoral immune responses to SARS‐CoV‐2 infection and vaccination. Clin & Trans Imm. 2022;11(3). 10.1002/cti2.1380 PMC8942165 35356067

[33] Bager MD, Tan FCC, Boisen MK, Krog SM, Nolsoee R, Collatz Christensen H, et al. Behavioral factors associated with SARS-CoV-2 infection. Results from a web-based case-control survey in the Capital Region of Denmark. BMJ Open. 2022;12(6):e056393. doi: 10.1136/bmjopen-2021-056393 PMC9170796 36691250

[34] de Lusignan S, Dorward J, Correa A, Jones N, Akinyemi O, Amirthalingam G, et al. Risk factors for SARS-CoV-2 among patients in the Oxford royal college of general practitioners research and surveillance centre primary care network: a cross-sectional study. Lancet Infect Dis. 2020;20(9):1034–42. doi: 10.1016/S1473-3099(20)30371-6 32422204 PMC7228715

[35] Hu S, Wang W, Wang Y, Litvinova M, Luo K, Ren L, et al. Infectivity, susceptibility, and risk factors associated with SARS-CoV-2 transmission under intensive contact tracing in Hunan, China. Nat Commun. 2021;12(1):1533. doi: 10.1038/s41467-021-21710-6 33750783 PMC7943579

[36] Ng OT, Marimuthu K, Koh V, Pang J, Linn KZ, Sun J, et al. SARS-CoV-2 seroprevalence and transmission risk factors among high-risk close contacts: a retrospective cohort study. Lancet Infect Dis. 2021;21(3):333–43. doi: 10.1016/S1473-3099(20)30833-1 33152271 PMC7831879

[37] Strathdee SA, Abramovitz D, Harvey-Vera A, Vera CF, Rangel G, Artamonova I, et al. Prevalence and correlates of SARS-CoV-2 seropositivity among people who inject drugs in the San Diego-Tijuana border region. PLoS One. 2021;16(11):e0260286. doi: 10.1371/journal.pone.0260286 34807963 PMC8608290

[38] Thomas DR, Fina LH, Adamson JP, Sawyer C, Jones A, Nnoaham K. Social, demographic and behavioural determinants of SARS-CoV-2 infection: a case-control study carried out during mass community testing of asymptomatic individuals in South Wales, December 2020. Epidemiol Infect. 2022;150:e115. 10.1017/S0950268822000620 PMC9203360 35535456

[39] Li Y, Wang X, Nair H. Global seasonality of human seasonal coronaviruses: a clue for postpandemic circulating season of severe acute respiratory syndrome coronavirus 2?. J Infect Dis. 2020. doi: jiaa436 10.1093/infdis/jiaa436 PMC7454715 32691843

[40] Hicks J, Klumpp-Thomas C, Kalish H, Shunmugavel A, Mehalko J, Denson JP. Serologic cross-reactivity of SARS-CoV-2 with endemic and seasonal Betacoronaviruses. medRxiv. 2020. doi: 10.1101/2020.06.22.20137695 PMC7962425 33725211

[41] Novelli S, Reinkemeyer C, Bulaev D, O’Sullivan MP, Snoeck CJ, Rauschenberger A, et al. Waning of anti-SARS-CoV-2 antibodies after the first wave of the COVID-19 pandemic in 2020: a 12-month-evaluation in three population-based European studies. PLoS One. 2025;20(5):e0320196. doi: 10.1371/journal.pone.0320196 40344145 PMC12063904

