# Supplementary material for: SARS-CoV-2 infection in female sex workers from Nairobi, Kenya early in the COVID-19 pandemic: Seroincidence and behavioural associations
Source: PLoS One. 2026 Jun 15;21(6):e0327692. doi: 10.1371/journal.pone.0327692 (PMC13268201; doi:10.1371/journal.pone.0327692)
Supplement: Attachment — Submitted filename: SARS-CoV-2 in FSWs in Nairobi.docx [file pone.0327692.s002.docx]

**­­­­­­­­­­KENYA MEDICAL RESEARCH INSTITUTE**

Center for Infectious and Diseases Control Research P.O. Box 3, BUSIA 50400, Kenya,

Tel: (055) 22232, Fax: 22410, Email: info@alupe-kemri.org Website: [www.kemri.org](http://www.kemri.org)

Seroepidemiological study of SARS-CoV-2 among female sex workers (FSWs) in Nairobi

**November 12, 2025**

**Reviewers report**

This manuscript describes a longitudinal seroepidemiological study of SARS-CoV-2 among female sex workers (FSWs) in Nairobi, Kenya. The authors report a high seroincidence early in the pandemic and assess associations between infection and socio-behavioural factors. The study addresses a relevant, under-researched population and adds important epidemiologic data. This is a well written manuscript, even though substantial improvements are needed in the justification of methods, transparency of statistical reporting, clarity of definitions, and compliance with ethical/data standards. Addressing these issues will significantly strengthen the manuscript.

Major Issues

1. Causal Inference and Confounding

The analysis identifies no behavioural or demographic associations with infection. I am somewhat surprised by this. But on further thinking, the observational design and limited adjustment for confounders (e.g., unmeasured non-sex work exposures, social and household mixing) surely undermine causal interpretations. This is a limitation. The authors should clearly state limitations regarding confounding and the inability to make causal inferences.

2. Serological Testing Limitations

Reliance solely on serological assays, without PCR confirmation, limits specificity for incident infection and can introduce uncertainty regarding timing. During the pandemic, serological tests were often notoriously inadequate. The manuscript should more robustly justify the serology methodology, when PCR tests were widely available, and point out this specific limitation in detecting acute or recent infections.

3. Statistical Methods and Power

While the manuscript references post-hoc corrections for multiple testing, reporting lacks detail and results tables do not always clarify which findings remained robust after correction. Null results are not accompanied by formal sensitivity or power analyses, raising concerns about undetected moderate associations. Is there something that can be done about that?

4. Ethical and Data Availability Compliance

The current data availability statement (“upon request”) does not fully comply with PLOS ONE’s data sharing policy. What are the specific data sharing and repository plans that the authors have. Or at least could you transparently state justified restrictions?

I also found some minor Issues that could be addressed to improve this manuscript.

1. Definitions and Clarity

The distinction between “seroincident” and “seroprevalent” cases, and categorization of symptomatic disease, are operationally vague or inconsistently described. More precise definitions are needed for reproducibility. To be honest, I am not so familiar with “sero-incident’.

2. Tables and Supplementary Data

Several tables reference ambiguous or unclear variable names (e.g., “TITLE of sleeping rooms in home mean”). All table and variable names should be reviewed for clarity.

3. Contextualization and References

Contextualization could be improved by citing broader literature on COVID-19 risk in key populations, particularly FSWs in low- and middle-income countries.

4. Language and Copyediting

The manuscript contains minor typographical and language errors, with some awkward phrasing. A final round of careful editing will enhance clarity and readability.

Recommendation

Major revision.

Prof. Matilu Mwau, M.B., Ch.B., MTM, D.Phil (Oxon), OGW
