# Supplementary material for: SARS-CoV-2 infection in female sex workers from Nairobi, Kenya early in the COVID-19 pandemic: Seroincidence and behavioural associations
Source: PLoS One. 2026 Jun 15;21(6):e0327692. doi: 10.1371/journal.pone.0327692 (PMC13268201; doi:10.1371/journal.pone.0327692)
Supplement: Attachment — Submitted filename: Response to Reviewers.docx [file pone.0327692.s003.docx]

January 23, 2026

Re: manuscript “SARS-CoV-2 infection in female sex workers from Nairobi, Kenya early in the COVID-19 pandemic: seroincidence and behavioural associations” (PONE-D-25-32697)

Dear Dr. Uzairue,

On the behalf of my coauthors, I would like to sincerely thank the editorial board and peer reviewers for their thoughtful review of the above-named manuscript.

As requested by all three of our reviewers, we have expanded our Introduction to highlight the novelty of this study (lines 73-75, 82-84), our Discussion section to further the dialogue on the real-life implications of this research (lines 405-418), as well as our limitations section to address key points we had missed in our original submission (lines 389-400). Importantly, we have updated our figures and tables for easier comprehension and made some minor changes to the language throughout the manuscript.

In addition, we have addressed editor’s journal requirement prompts, as follows:

- The PLOS inclusivity questionnaire has been completed and included as Supporting Information (S1 File)
- All funding information has been removed from the manuscript and our updated funding statement is as follows:

“Canadian Institutes of Health Research (CIHR; RK #MM1-174919); RK is supported by the Elisabeth Hofmann Chair in Translational Research. ELISA assays were performed at the Network Biology Collaborative Centre (RRID: SCR_025390) at the Lunenfeld-Tanenbaum Research Institute, a facility supported by Canada Foundation for Innovation funding, by the Government of Ontario and by Genome Canada and Ontario Genomics (OGI-139). Anne-Claude Gingras is the Canada Research Chair in Functional Proteomics.”

- We would like to update our financial disclosures as follows:

“Canadian Institutes of Health Research: Institute of Infection and Immunity (CIHR; RK #MM1-174919); RK is supported by the Elisabeth Hofmann Chair in Translational Research. ELISA assays were performed at the Network Biology Collaborative Centre (RRID: SCR_025390) at the Lunenfeld-Tanenbaum Research Institute, a facility supported by Canada Foundation for Innovation funding, by the Government of Ontario and by Genome Canada and Ontario Genomics (OGI-139). Anne-Claude Gingras holds the Canada Research Chair in Functional Proteomics. The funders had no role in study design, data collection and analysis, decision to publish, or preparation of the manuscript.”

- Regarding data availability, we are not able to publicly deposit data from this study as our REB feels that this could breach protection of participant confidentiality and safety. We have updated our data availability statement as follows:

“ The data are not publicly available due to the need to protect participant confidentiality and safety. Our commitment to participant confidentiality and safety is detailed in our participant information sheet and consent forms, as approved by the Kenyatta National Hospital ERB (P778/11/2018 and P514/06/2024) and the University of Toronto REB (protocols #37046 and REB#41580). Requests to access data should be directed to the University of Toronto Research Ethics Board at: ethics.review@utoronto.ca”

- We would like to clarify that figure 2 is a map created by the first author, and the author therefore owns the image and the copyright. There is no previously copyrighted figure.
- In regard to Datawrapper, the author of this manuscript has been given permission from the company to share an email thread confirming the copyright as they do not have copyright permissions explicitly stated on their website. Please let us know which the best way is to share this email thread.
- The map data source from OCHA can be found here: <https://data.humdata.org/dataset/cod-ab-ken>
- The copyright licensing terms for this data source can be found here: <https://data.humdata.org/faqs/licenses#:~:text=Creative%20Commons%20Attribution%20for%20Intergovernmental%20Organisations%20(CC%20BY-IGO)>

We believe that these revisions, outlined in detail in the point-by-point response below, have substantially improved the manuscript. We hope that the editorial board and peer reviewers agree, and that the manuscript is now acceptable for publication in PLOS ONE.

Sincerely,

Su Yang

-----------------------------------------------------------

**Reviewer #1:**

• Novelty and Significance:

The study’s focus on FSWs is highly relevant and novel for the region, but the introduction should more clearly articulate what is new compared to other African serosurveys (e.g., truck drivers, informal settlements). The policy relevance of the findings—how they inform pandemic preparedness for key populations—should be elaborated in the discussion.

**We have revised the Introduction to more clearly distinguish what is new in this serosurvey, specifically how this manuscript is the first to describe SARS-CoV-2 infection in Kenyan sex workers (lines 73-74, 82-87). As well we have elaborated on the relevance of our findings in the Discussion (lines 405-411).**

• Methods:

The time frame for data collection (June 2020–February 2021) spans multiple transmission phases. Please clarify whether analyses adjusted for temporal variation in exposure risk. Six 'pre-pandemic positive' samples were excluded. Provide a rationale and consider a sensitivity analysis including these to show their influence on incidence estimates. Indicate whether multiple comparisons adjustments were applied for behavioural variables. Consider including a flow diagram summarizing recruitment, exclusions, and analytical sample size.

**Analyses were not adjusted for time since start of the pandemic, as this was a parameter we included in our analysis. We have included a line to explain this in our Methods (lines 175-177).**

**Rationale for exclusion of the 6 samples can now be found in the Results section (lines 213-215).**

**Clarifications for the application of multiple comparison have now been added to the Methods (lines 173-174).**

**Our figure 1 has been adjusted to greater reflect reviewer 1’s comments and is referenced in the manuscript (lines 183-186, 216)(Figure 1)**

• Results:

The presentation is clear, but results could be better contextualized. For example, the lack of association between infection and behavioural characteristics warrants interpretation—was exposure more community-driven than occupation-specific? The high rate of self-reported symptoms among seronegative participants requires discussion—possible explanations could include recall bias or other respiratory pathogens. Consider shortening subheadings and restructuring for clarity.

**We thank the reviewer for these suggestions, and we have now included a more nuanced interpretation of our findings in the Discussion (lines 373-377). Possible explanations of our high symptom recall have been expanded in the limitation of our work (lines 263-264, 344-345, 347-349).**

• Discussion and Interpretation:

Expand on the public health implications of findings: how should policymakers and global health actors use this evidence to improve inclusion of FSWs in surveillance and vaccination programs? Provide a deeper explanation of why expected risk differentials were not observed. End the discussion with a forward-looking paragraph linking lessons learned to pandemic preparedness, equity, and surveillance integration for key populations.

**This is an excellent suggestion. We have now expanded our Discussion to include dialogue on the public health implications of our findings (lines 404-414).**

• Limitations:

Strengthen the limitations section by acknowledging: possible misclassification due to antibody waning or sensitivity variation; recall bias from self-reported symptoms; and limited generalizability beyond Nairobi FSWs.

**These factors have been added to our limitations section (lines 388-395).**

**Reviewer #2:**

Reviewers report:

This manuscript describes a longitudinal seroepidemiological study of SARS-CoV-2 among female sex workers (FSWs) in Nairobi, Kenya. The authors report a high seroincidence early in the pandemic and assess associations between infection and socio-behavioural factors. The study addresses a relevant, under-researched population and adds important epidemiologic data. This is a well written manuscript, even though substantial improvements are needed in the justification of methods, transparency of statistical reporting, clarity of definitions, and compliance with ethical/data standards. Addressing these issues will significantly strengthen the manuscript.

**We appreciate the time taken by the reviewer to give us such a thorough review.**

Major Issues

1. Causal Inference and Confounding

The analysis identifies no behavioural or demographic associations with infection. I am somewhat surprised by this. But on further thinking, the observational design and limited adjustment for confounders (e.g., unmeasured non-sex work exposures, social and household mixing) surely undermine causal interpretations. This is a limitation. The authors should clearly state limitations regarding confounding and the inability to make causal inferences.

**We have updated our limitations sections to address confounding and the inability to make causal inferences per the reviewer’s suggestions (lines 373-377 and lines 395-397).**

2. Serological Testing Limitations

Reliance solely on serological assays, without PCR confirmation, limits specificity for incident infection and can introduce uncertainty regarding timing. During the pandemic, serological tests were often notoriously inadequate. The manuscript should more robustly justify the serology methodology, when PCR tests were widely available, and point out this specific limitation in detecting acute or recent infections.

**Serological testing was chosen for practical reasons. Most importantly, we received feedback from the community that they would not participate in a study that involved mandatory PCR testing, as there was government-mandated quarantine and so a positive test would have major financial implications. We have now formally addressed this in our methods (lines 137-141) and limitations (line 388-389).**

3. Statistical Methods and Power

While the manuscript references post-hoc corrections for multiple testing, reporting lacks detail and results tables do not always clarify which findings remained robust after correction. Null results are not accompanied by formal sensitivity or power analyses, raising concerns about undetected moderate associations. Is there something that can be done about that?

**We thank the reviewer for raising these concerns. We have added clarifying details to our Tables 3-5 to make it clear that p-values were unadjusted, and we have reworded our statistical methods to make the exploratory nature of our analysis more apparent to readers (lines 173-174, 370-371).**

**Although the reviewer is correct that weaker associations may have been missed, a sample size of 1000 is not trivial. We have now addressed concerns about undetected moderate associations within our limitations section (lines 398-399).**

4. Ethical and Data Availability Compliance

The current data availability statement (“upon request”) does not fully comply with PLOS ONE’s data sharing policy. What are the specific data sharing and repository plans that the authors have. Or at least could you transparently state justified restrictions?

**Data availability statement has been updated to address the data sharing restriction unique to our study.**

I also found some minor Issues that could be addressed to improve this manuscript.

1. Definitions and Clarity

The distinction between “seroincident” and “seroprevalent” cases, and categorization of symptomatic disease, are operationally vague or inconsistently described. More precise definitions are needed for reproducibility. To be honest, I am not so familiar with “sero-incident’.

**We appreciate the concerns raised about use of “seroincident” and “seroprevalent”. The terms are now used more carefully throughout the manuscript, and we hope that this is now more clear.**

2. Tables and Supplementary Data

Several tables reference ambiguous or unclear variable names (e.g., “TITLE of sleeping rooms in home mean”). All table and variable names should be reviewed for clarity.

**All tables have been reviewed for clarity, in refences to the example variable we have changed the “#” symbol to “number” to avoid any confusion in Tables 1, 3-5.**

3. Contextualization and References

Contextualization could be improved by citing broader literature on COVID-19 risk in key populations, particularly FSWs in low- and middle-income countries.

**We had now added several references that expand on COVID-19 risk in FSWs in our Introduction (lines 79-87)**

4. Language and Copyediting

The manuscript contains minor typographical and language errors, with some awkward phrasing. A final round of careful editing will enhance clarity and readability.

**We thank the reviewer for this suggestion. We have done a thorough round of editing on the entirety of the manuscript to improve readability and try to reduce awkward phrasing (various lines)**

Recommendation

Major revision

**Reviewer #3:**

This is a well written and straightforward manuscript characterizing the seroincidence of SARS-CoV-2 infection early in the COVID-19 pandemic in a cohort of female sex workers in Nairobi, Kenya. The manuscript is clearly written and provides data that adds to the published literature describing the high seroincidence and low symptomatic rate of SARS-CoV-2 infection in Sub-Saharan Africa during the pandemic. Surprisingly, the manuscript did not identify any behavioral or demographic factors associated with seroincidence in this cohort.

There are a few revisions that would improve the paper:

• The authors report that the seroincidence of 27.7% in this FSW cohort was not higher than the general population - which they cite as increasing from 4.3% in 2020 to 48.5% in 2021. However, the sample collection for this FSW cohort was limited to followup samples collected June 2020-February 2021, and it needs to be a bit more clearly stated in the intro and discussion the exact timing of the seroincidence rates of other populations they are comparing to – i.e. what month in 2021 does the seroincidence rate reflect in the other populations (without asking the reader to look at the references).

**We thank the reviewer for this comment and have made changes to both our Introduction and Discussion to make more concise comparisons of seroincidence rates between our cohort and other population at the same time point (lines 65,69, 330-332).**

• Baseline samples were collected between June-Dec 2019. Follow up samples that were tested were collected between June 2020-February 2021, a 7-month window. The authors note the mean of 189 days between start of pandemic and follow-up visit date in the text and at the bottom of Table 3. This would be better if it was a median and regardless of median or mean – the range needs to also be reported in both the text and in Table 3 - and the authors should more clearly state the caveat that some individuals were only followed for a few months, and thus the seroincidence reported is inevitably an underestimate of the true seroincidence in this cohort by Feb 2021. The data would be more accurate if there was a sample from everyone from Jan-Feb 2021, but this is clearly not possible at this point, so the limitation should be clearly stated, and the data in the supplemental table should described in the resutls section and be moved to the main text to show the higher rate in the group sampled later.

**The median of follow-up visit dates is now reported, instead of mean, in Table 3 as well as in text (lines 35, 223).**

**We have added language in the limitations section of this manuscript that states possible underestimation of true seroincidence due to short follow up periods (lines 390-392).**

**Additionally, as suggested by the reviewer, we have moved the supplemental table into the main text and results section (lines 233-242) (Table 4).**

• This reviewer appreciates the use of more than one antigen to define seropositive status and the reporting of the false-positives with only 1 antigen detected. Would be good to comment on this in the discussion noting that RBD had the lowest background - and also the reasons that only 2 of the 3 antigens are detected in ~10% of samples.

**As suggested by the reviewer, we have expanded our Discussion section to address the false-positive rate and the low background levels of RBD antibodies (lines 378-387), as well as the fact that baseline presence of antibodies against one antigen was not protective against subsequent infection.**

• However, the authors then use the single-positives as a measure of those with pre-pandemic antibodies to SARS-CoV-2 as stated on Page 13 and Table 4. If they are suggesting that these are false positives due to the presence of cross-reactive antibodies to other infections (possibly related coronaviruses, etc..) then they should state that as such. Otherwise it seems odd to use the false positives from an assay to measure the potential of pre-existing immunity.

**False-positives in our pre-pandemic samples were again defined as having antibodies above the antigen specific threshold for 2 out of 3 antigens. We have changed our language to make this clearer in several sections including the Results (lines 209-210).**

• The authors should carefully review all of the numbers in the text compared to the tables and Figure 1. There appear to be discrepancies. For example:

o Table 2 shows 827 baseline samples included and 229 seropositive while Figure 1 shows 822 included with 226 seropositive. Table 3 shows 226 seroincident and 595 seronegative. Even with the 6 false positives removed the numbers don’t match (229-6 = 223).

**We thank the reviewer for the careful review of our numbers, we have fixed any discrepancies with numbers in Table 1 and Table 2, and have redesigned our Figure 1 chart to make the numbers clearer (Figure 1).**

o Numbers of “Antibodies at baseline” in Table 4 does not match the data shown in Table 2 or the text. Antibodies at baseline against ANY of the 3 antigens in Table4 = 35+91=126 while Table 2 shows 6+61+2+82=151. Abs against spike = 12+43=55 in Table 4 vs 61 in Table 2, etc… Please check all the numbers in the paper as i may have missed some discrepancies.

**We would like to clarify that all participants with samples available for serology were included in Table 2, however Table 4 included only those with both baseline and follow-up samples hence the differences in reported numbers. We have included language in the results to make this distinction clear (lines 248-250).**
