# Supplementary material for: SARS-CoV-2 infection in female sex workers from Nairobi, Kenya early in the COVID-19 pandemic: Seroincidence and behavioural associations
Source: PLoS One. 2026 Jun 15;21(6):e0327692. doi: 10.1371/journal.pone.0327692 (PMC13268201; doi:10.1371/journal.pone.0327692)
Supplement: Peer review history

# Peer review: Decision Letter 0

**DOI:** 10.1371/journal.pone.0327692.r001

**Authors:** Leonard Uzairue

10 Dec 2025

PONE-D-25-32697SARS-CoV-2 infection in female sex workers from Nairobi, Kenya early in the COVID-19 pandemic: seroincidence and behavioural associations.PLOS One

Dear Dr. Yang,

Thank you for submitting your manuscript to PLOS ONE. After careful consideration, we feel that it has merit but does not fully meet PLOS ONE’s publication criteria as it currently stands. Therefore, we invite you to submit a revised version of the manuscript that addresses the points raised during the review process.

Please submit your revised manuscript by Jan 24 2026 11:59PM. If you will need more time than this to complete your revisions, please reply to this message or contact the journal office at plosone@plos.org. When you're ready to submit your revision, log on to https://www.editorialmanager.com/pone/ and select the 'Submissions Needing Revision' folder to locate your manuscript file.

Please include the following items when submitting your revised manuscript:

- A rebuttal letter that responds to each point raised by the academic editor and reviewer(s). You should upload this letter as a separate file labeled 'Response to Reviewers'.
- A marked-up copy of your manuscript that highlights changes made to the original version. You should upload this as a separate file labeled 'Revised Manuscript with Track Changes'.
- An unmarked version of your revised paper without tracked changes. You should upload this as a separate file labeled 'Manuscript'.

If you would like to make changes to your financial disclosure, please include your updated statement in your cover letter. Guidelines for resubmitting your figure files are available below the reviewer comments at the end of this letter.

If applicable, we recommend that you deposit your laboratory protocols in protocols.io to enhance the reproducibility of your results. Protocols.io assigns your protocol its own identifier (DOI) so that it can be cited independently in the future. For instructions see: https://journals.plos.org/plosone/s/submission-guidelines#loc-laboratory-protocols. Additionally, PLOS ONE offers an option for publishing peer-reviewed Lab Protocol articles, which describe protocols hosted on protocols.io. Read more information on sharing protocols at https://plos.org/protocols?utm_medium=editorial-email&utm_source=authorletters&utm_campaign=protocols.

We look forward to receiving your revised manuscript.

Kind regards,

Leonard Ighodalo Uzairue, PhD

Academic Editor

PLOS One

Journal requirements:

When submitting your revision, we need you to address these additional requirements.

1. Please ensure that your manuscript meets PLOS ONE's style requirements, including those for file naming. The PLOS ONE style templates can be found at

https://journals.plos.org/plosone/s/file?id=wjVg/PLOSOne_formatting_sample_main_body.pdf and

https://journals.plos.org/plosone/s/file?id=ba62/PLOSOne_formatting_sample_title_authors_affiliations.pdf

2. Please include a complete copy of PLOS’ questionnaire on inclusivity in global research in your revised manuscript. Our policy for research in this area aims to improve transparency in the reporting of research performed outside of researchers’ own country or community. The policy applies to researchers who have travelled to a different country to conduct research, research with Indigenous populations or their lands, and research on cultural artefacts. The questionnaire can also be requested at the journal’s discretion for any other submissions, even if these conditions are not met. Please find more information on the policy and a link to download a blank copy of the questionnaire here: https://journals.plos.org/plosone/s/best-practices-in-research-reporting. Please upload a completed version of your questionnaire as Supporting Information when you resubmit your manuscript.

3. Thank you for stating the following in the Acknowledgments Section of your manuscript:

“Canadian Institutes of Health Research (CIHR; RK #MM1-174919); RK is supported by the Elisabeth Hofmann Chair in Translational Research.”

We note that you have provided funding information that is not currently declared in your Funding Statement. However, funding information should not appear in the Acknowledgments section or other areas of your manuscript. We will only publish funding information present in the Funding Statement section of the online submission form.

Please remove any funding-related text from the manuscript and let us know how you would like to update your Funding Statement. Currently, your Funding Statement reads as follows:

“The author(s) received no specific funding for this work.”

Please include your amended statements within your cover letter; we will change the online submission form on your behalf.

4. We note that the grant information you provided in the ‘Funding Information’ and ‘Financial Disclosure’ sections do not match.

When you resubmit, please ensure that you provide the correct grant numbers for the awards you received for your study in the ‘Funding Information’ section.

5. Thank you for stating the following financial disclosure:

“The author(s) received no specific funding for this work.”

At this time, please address the following queries:

a) Please clarify the sources of funding (financial or material support) for your study. List the grants or organizations that supported your study, including funding received from your institution.

b) State what role the funders took in the study. If the funders had no role in your study, please state: “The funders had no role in study design, data collection and analysis, decision to publish, or preparation of the manuscript.”

c) If any authors received a salary from any of your funders, please state which authors and which funders.

d) If you did not receive any funding for this study, please state: “The authors received no specific funding for this work.”

Please include your amended statements within your cover letter; we will change the online submission form on your behalf.

6. In the online submission form, you indicated that “Data cannot be shared publicly due to privacy concerns as sex worker participants may be identifiable from socio-behavioural parameters. De-identified data sets will be made available upon request.”

All PLOS journals now require all data underlying the findings described in their manuscript to be freely available to other researchers, either 1. In a public repository, 2. Within the manuscript itself, or 3. Uploaded as supplementary information.

This policy applies to all data except where public deposition would breach compliance with the protocol approved by your research ethics board. If your data cannot be made publicly available for ethical or legal reasons (e.g., public availability would compromise patient privacy), please explain your reasons on resubmission and your exemption request will be escalated for approval.

7. Please include a caption for figure 1.

8. Please ensure that you refer to Figure 1 in your text as, if accepted, production will need this reference to link the reader to the figure.

9. We note that Figure 2 in your submission contain map images which may be copyrighted. All PLOS content is published under the Creative Commons Attribution License (CC BY 4.0), which means that the manuscript, images, and Supporting Information files will be freely available online, and any third party is permitted to access, download, copy, distribute, and use these materials in any way, even commercially, with proper attribution. For these reasons, we cannot publish previously copyrighted maps or satellite images created using proprietary data, such as Google software (Google Maps, Street View, and Earth). For more information, see our copyright guidelines: http://journals.plos.org/plosone/s/licenses-and-copyright.

We require you to either (1) present written permission from the copyright holder to publish these figures specifically under the CC BY 4.0 license, or (2) remove the figures from your submission:

1. You may seek permission from the original copyright holder of Figure(s) [#] to publish the content specifically under the CC BY 4.0 license.

We recommend that you contact the original copyright holder with the Content Permission Form (http://journals.plos.org/plosone/s/file?id=7c09/content-permission-form.pdf) and the following text:

“I request permission for the open-access journal PLOS ONE to publish XXX under the Creative Commons Attribution License (CCAL) CC BY 4.0 (http://creativecommons.org/licenses/by/4.0/).

Please be aware that this license allows unrestricted use and distribution, even commercially, by third parties. Please reply and provide explicit written permission to publish XXX under a CC BY license and complete the attached form.”

Please upload the completed Content Permission Form or other proof of granted permissions as an "Other" file with your submission.

In the figure caption of the copyrighted figure, please include the following text: “Reprinted from [ref] under a CC BY license, with permission from [name of publisher], original copyright [original copyright year].”

2. If you are unable to obtain permission from the original copyright holder to publish these figures under the CC BY 4.0 license or if the copyright holder’s requirements are incompatible with the CC BY 4.0 license, please either i) remove the figure or ii) supply a replacement figure that complies with the CC BY 4.0 license. Please check copyright information on all replacement figures and update the figure caption with source information. If applicable, please specify in the figure caption text when a figure is similar but not identical to the original image and is therefore for illustrative purposes only.

The following resources for replacing copyrighted map figures may be helpful:

USGS National Map Viewer (public domain): http://viewer.nationalmap.gov/viewer/

The Gateway to Astronaut Photography of Earth (public domain): http://eol.jsc.nasa.gov/sseop/clickmap/

Maps at the CIA (public domain): https://www.cia.gov/library/publications/the-world-factbook/index.html and https://www.cia.gov/library/publications/cia-maps-publications/index.html

NASA Earth Observatory (public domain): http://earthobservatory.nasa.gov/

Landsat: http://landsat.visibleearth.nasa.gov/

USGS EROS (Earth Resources Observatory and Science (EROS) Center) (public domain): http://eros.usgs.gov/#

Natural Earth (public domain): http://www.naturalearthdata.com/

10. Please include your tables as part of your main manuscript and remove the individual files. Please note that supplementary tables (should remain/ be uploaded) as separate "supporting information" files.

11. Please include captions for your Supporting Information files at the end of your manuscript, and update any in-text citations to match accordingly. Please see our Supporting Information guidelines for more information: http://journals.plos.org/plosone/s/supporting-information.

[Note: HTML markup is below. Please do not edit.]

Reviewers' comments:

Reviewer's Responses to Questions

**Comments to the Author**

1. Is the manuscript technically sound, and do the data support the conclusions?

The manuscript must describe a technically sound piece of scientific research with data that supports the conclusions. Experiments must have been conducted rigorously, with appropriate controls, replication, and sample sizes. The conclusions must be drawn appropriately based on the data presented.

Reviewer #1: Yes

Reviewer #2: Yes

Reviewer #3: Yes

**********

2. Has the statistical analysis been performed appropriately and rigorously?

Reviewer #1: Yes

Reviewer #2: Yes

Reviewer #3: Yes

**********

3. Have the authors made all data underlying the findings in their manuscript fully available?

The PLOS Data policy requires authors to make all data underlying the findings described in their manuscript fully available without restriction, with rare exception (please refer to the Data Availability Statement in the manuscript PDF file). The data should be provided as part of the manuscript or its supporting information, or deposited to a public repository. For example, in addition to summary statistics, the data points behind means, medians and variance measures should be available. If there are restrictions on publicly sharing data—e.g. participant privacy or use of data from a third party—those must be specified.

Reviewer #1: No

Reviewer #2: No

Reviewer #3: No

**********

4. Is the manuscript presented in an intelligible fashion and written in standard English?

PLOS ONE does not copyedit accepted manuscripts, so the language in submitted articles must be clear, correct, and unambiguous. Any typographical or grammatical errors should be corrected at revision, so please note any specific errors here.

Reviewer #1: Yes

Reviewer #2: Yes

Reviewer #3: Yes

**********

5. Review Comments to the Author

Please use the space provided to explain your answers to the questions above. You may also include additional comments for the author, including concerns about dual publication, research ethics, or publication ethics. (Please upload your review as an attachment if it exceeds 20,000 characters)

Reviewer #1: • Novelty and Significance:

The study’s focus on FSWs is highly relevant and novel for the region, but the introduction should more clearly articulate what is new compared to other African serosurveys (e.g., truck drivers, informal settlements). The policy relevance of the findings—how they inform pandemic preparedness for key populations—should be elaborated in the discussion.

• Methods:

The time frame for data collection (June 2020–February 2021) spans multiple transmission phases. Please clarify whether analyses adjusted for temporal variation in exposure risk. Six 'pre-pandemic positive' samples were excluded. Provide a rationale and consider a sensitivity analysis including these to show their influence on incidence estimates. Indicate whether multiple comparisons adjustments were applied for behavioural variables. Consider including a flow diagram summarizing recruitment, exclusions, and analytical sample size.

• Results:

The presentation is clear, but results could be better contextualized. For example, the lack of association between infection and behavioural characteristics warrants interpretation—was exposure more community-driven than occupation-specific? The high rate of self-reported symptoms among seronegative participants requires discussion—possible explanations could include recall bias or other respiratory pathogens. Consider shortening subheadings and restructuring for clarity.

• Discussion and Interpretation:

Expand on the public health implications of findings: how should policymakers and global health actors use this evidence to improve inclusion of FSWs in surveillance and vaccination programs? Provide a deeper explanation of why expected risk differentials were not observed. End the discussion with a forward-looking paragraph linking lessons learned to pandemic preparedness, equity, and surveillance integration for key populations.

• Limitations:

Strengthen the limitations section by acknowledging: possible misclassification due to antibody waning or sensitivity variation; recall bias from self-reported symptoms; and limited generalizability beyond Nairobi FSWs.

Reviewer #2: Seroepidemiological study of SARS-CoV-2 among female sex workers (FSWs) in Nairobi

November 12, 2025

Reviewers report

This manuscript describes a longitudinal seroepidemiological study of SARS-CoV-2 among female sex workers (FSWs) in Nairobi, Kenya. The authors report a high seroincidence early in the pandemic and assess associations between infection and socio-behavioural factors. The study addresses a relevant, under-researched population and adds important epidemiologic data. This is a well written manuscript, even though substantial improvements are needed in the justification of methods, transparency of statistical reporting, clarity of definitions, and compliance with ethical/data standards. Addressing these issues will significantly strengthen the manuscript.

Major Issues

1. Causal Inference and Confounding

The analysis identifies no behavioural or demographic associations with infection. I am somewhat surprised by this. But on further thinking, the observational design and limited adjustment for confounders (e.g., unmeasured non-sex work exposures, social and household mixing) surely undermine causal interpretations. This is a limitation. The authors should clearly state limitations regarding confounding and the inability to make causal inferences.

2. Serological Testing Limitations

Reliance solely on serological assays, without PCR confirmation, limits specificity for incident infection and can introduce uncertainty regarding timing. During the pandemic, serological tests were often notoriously inadequate. The manuscript should more robustly justify the serology methodology, when PCR tests were widely available, and point out this specific limitation in detecting acute or recent infections.

3. Statistical Methods and Power

While the manuscript references post-hoc corrections for multiple testing, reporting lacks detail and results tables do not always clarify which findings remained robust after correction. Null results are not accompanied by formal sensitivity or power analyses, raising concerns about undetected moderate associations. Is there something that can be done about that?

4. Ethical and Data Availability Compliance

The current data availability statement (“upon request”) does not fully comply with PLOS ONE’s data sharing policy. What are the specific data sharing and repository plans that the authors have. Or at least could you transparently state justified restrictions?

I also found some minor Issues that could be addressed to improve this manuscript.

1. Definitions and Clarity

The distinction between “seroincident” and “seroprevalent” cases, and categorization of symptomatic disease, are operationally vague or inconsistently described. More precise definitions are needed for reproducibility. To be honest, I am not so familiar with “sero-incident’.

2. Tables and Supplementary Data

Several tables reference ambiguous or unclear variable names (e.g., “TITLE of sleeping rooms in home mean”). All table and variable names should be reviewed for clarity.

3. Contextualization and References

Contextualization could be improved by citing broader literature on COVID-19 risk in key populations, particularly FSWs in low- and middle-income countries.

4. Language and Copyediting

The manuscript contains minor typographical and language errors, with some awkward phrasing. A final round of careful editing will enhance clarity and readability.

Recommendation

Major revision.

Reviewer #3: This is a well written and straightforward manuscript characterizing the seroincidence of SARS-CoV-2 infection early in the COVID-19 pandemic in a cohort of female sex workers in Nairobi, Kenya. The manuscript is clearly written and provides data that adds to the published literature describing the high sero-incidence and low symptomatic rate of SARS-CoV-2 infection in sub-saharan Africa during the pandemic. Surprisingly, the manuscript did not identify any behavioral or demographic factors associated with seroincidence in this cohort.

There are a few revisions that would improve the paper:

• The authors report that the sero-incidence of 27.7% in this FSW cohort was not higher than the general population - which they cite as increasing from 4.3% in 2020 to 48.5% in 2021. However, the sample collection for this FSW cohort was limited to followup samples collected June 2020-February 2021, and it needs to be a bit more clearly stated in the intro and discussion the exact timing of the seroincidence rates of other populations they are comparing to – i.e. what month in 2021 does the seroincidence rate reflect in the other populations (without asking the reader to look at the references).

• Baseline samples were collected between June-Dec 2019. Follow up samples that were tested were collected between June 2020-February 2021, a 7 month window. The authors note the mean of 189 days between start of pandemic and followup visit date in the text and at the bottom of Table 3. This would be better if it was a median and regardless of median or mean – the range needs to also be reported in both the text and in Table 3 - and the authors should more clearly state the caveat that some individuals were only followed for a few months, and thus the seroincidence reported is inevitably an underestimate of the true seroincidence in this cohort by Feb 2021. The data would be more accurate if there was a sample from everyone from Jan-Feb 2021, but this is clearly not possible at this point, so the limitation should be clearly stated, and the data in the supplemental table should described in the resutls section and be moved to the main text to show the higher rate in the group sampled later.

• This reviewer appreciates the use of more than one antigen to defline seropositive status and the reporting of the false-positives with only 1 antigen detected. Would be good to comment on this in the discussion noting that RBD had the lowest background - and also the reasons that only 2 of the 3 antigens are detected in ~10% of samples.

• However, the authors then use the single-positives as a measure of those with pre-pandemic antibodies to SARS-CoV-2 as stated on Page 13 and Table 4. If they are suggesting that these are false positives due to the presence of cross-reactive antibodies to other infections (possibly related coronaviruses, etc..) then they should state that as such. Otherwise it seems odd to use the false positives from an assay to measure the potential of pre-existing immunity.

• The authors should carefully review all of the numbers in the text compared to the tables and Figure 1. There appear to be discrepancies. For example:

o Table 2 shows 827 baseline samples included and 229 seropositive while Figure 1 shows 822 included with 226 seropositive. Table 3 shows 226 seroincident and 595 seronegative. Even with the 6 false positives removed the numbers don’t match (229-6 = 223).

o Numbers of “Antibodies at baseline” in Table 4 does not match the data shown in Table 2 or the text. Antibodies at baseline against ANY of the 3 antigens in Table4 = 35+91=126 while Table 2 shows 6+61+2+82=151. Abs against spike = 12+43=55 in Table 4 vs 61 in Table 2, etc… Please check all the numbers in the paper as i may have missed some discrepancies.

**********

6. PLOS authors have the option to publish the peer review history of their article (what does this mean?). If published, this will include your full peer review and any attached files.

If you choose “no”, your identity will remain anonymous but your review may still be made public.

**Do you want your identity to be public for this peer review?**  For information about this choice, including consent withdrawal, please see our Privacy Policy.

Reviewer #1: **Yes:**  Dr Adetunji Adedokun

Reviewer #2: No

Reviewer #3: No

**********

[NOTE: If reviewer comments were submitted as an attachment file, they will be attached to this email and accessible via the submission site. Please log into your account, locate the manuscript record, and check for the action link "View Attachments". If this link does not appear, there are no attachment files.]

To ensure your figures meet our technical requirements, please review our figure guidelines: https://journals.plos.org/plosone/s/figures

You may also use PLOS’s free figure tool, NAAS, to help you prepare publication quality figures: https://journals.plos.org/plosone/s/figures#loc-tools-for-figure-preparation.

NAAS will assess whether your figures meet our technical requirements by comparing each figure against our figure specifications.

Attachment Submitted filename: SARS-CoV-2 in FSWs in Nairobi.docx

# Author comment: Author response to Decision Letter 1

**DOI:** 10.1371/journal.pone.0327692.r002

23 Feb 2026

we have addressed editor’s journal requirement prompts, as follows:

- The PLOS inclusivity questionnaire has been completed and included as Supporting Information (S1 File)

- All funding information has been removed from the manuscript and our updated funding statement is as follows:

“Canadian Institutes of Health Research (CIHR; RK #MM1-174919); RK is supported by the Elisabeth Hofmann Chair in Translational Research. ELISA assays were performed at the Network Biology Collaborative Centre (RRID: SCR_025390) at the Lunenfeld-Tanenbaum Research Institute, a facility supported by Canada Foundation for Innovation funding, by the Government of Ontario and by Genome Canada and Ontario Genomics (OGI-139). Anne-Claude Gingras is the Canada Research Chair in Functional Proteomics.”

- We would like to update our financial disclosures as follows:

“Canadian Institutes of Health Research: Institute of Infection and Immunity (CIHR; RK #MM1-174919); RK is supported by the Elisabeth Hofmann Chair in Translational Research. ELISA assays were performed at the Network Biology Collaborative Centre (RRID: SCR_025390) at the Lunenfeld-Tanenbaum Research Institute, a facility supported by Canada Foundation for Innovation funding, by the Government of Ontario and by Genome Canada and Ontario Genomics (OGI-139). Anne-Claude Gingras holds the Canada Research Chair in Functional Proteomics. The funders had no role in study design, data collection and analysis, decision to publish, or preparation of the manuscript.”

- Regarding data availability, we are not able to publicly deposit data from this study as our REB feels that this could breach protection of participant confidentiality and safety. We have updated our data availability statement as follows:

“ The data are not publicly available due to the need to protect participant confidentiality and safety. Our commitment to participant confidentiality and safety is detailed in our participant information sheet and consent forms, as approved by the Kenyatta National Hospital ERB (P778/11/2018 and P514/06/2024) and the University of Toronto REB (protocols #37046 and REB#41580). Requests to access data should be directed to the University of Toronto Research Ethics Board at: ethics.review@utoronto.ca”

- We would like to clarify that figure 2 is a map created by the first author, and the author therefore owns the image and the copyright. There is no previously copyrighted figure.

- In regard to Datawrapper, the author of this manuscript has been given permission from the company to share an email thread confirming the copyright as they do not have copyright permissions explicitly stated on their website. Please let us know which the best way is to share this email thread.

- The map data source from OCHA can be found here: https://data.humdata.org/dataset/cod-ab-ken

- The copyright licensing terms for this data source can be found here: https://data.humdata.org/faqs/licenses#:~:text=Creative%20Commons%20Attribution%20for%20Intergovernmental%20Organisations%20(CC%20BY-IGO)

We believe that these revisions, outlined in detail in the point-by-point response below, have substantially improved the manuscript. We hope that the editorial board and peer reviewers agree, and that the manuscript is now acceptable for publication in PLOS ONE.

Sincerely,

Su Yang

-----------------------------------------------------------

Reviewer #1:

• Novelty and Significance:

The study’s focus on FSWs is highly relevant and novel for the region, but the introduction should more clearly articulate what is new compared to other African serosurveys (e.g., truck drivers, informal settlements). The policy relevance of the findings—how they inform pandemic preparedness for key populations—should be elaborated in the discussion.

We have revised the Introduction to more clearly distinguish what is new in this serosurvey, specifically how this manuscript is the first to describe SARS-CoV-2 infection in Kenyan sex workers (lines 73-74, 82-87). As well we have elaborated on the relevance of our findings in the Discussion (lines 405-411).

• Methods:

The time frame for data collection (June 2020–February 2021) spans multiple transmission phases. Please clarify whether analyses adjusted for temporal variation in exposure risk. Six 'pre-pandemic positive' samples were excluded. Provide a rationale and consider a sensitivity analysis including these to show their influence on incidence estimates. Indicate whether multiple comparisons adjustments were applied for behavioural variables. Consider including a flow diagram summarizing recruitment, exclusions, and analytical sample size.

Analyses were not adjusted for time since start of the pandemic, as this was a parameter we included in our analysis. We have included a line to explain this in our Methods (lines 175-177).

Rationale for exclusion of the 6 samples can now be found in the Results section (lines 213-215).

Clarifications for the application of multiple comparison have now been added to the Methods (lines 173-174).

Our figure 1 has been adjusted to greater reflect reviewer 1’s comments and is referenced in the manuscript (lines 183-186, 216)(Figure 1)

• Results:

The presentation is clear, but results could be better contextualized. For example, the lack of association between infection and behavioural characteristics warrants interpretation—was exposure more community-driven than occupation-specific? The high rate of self-reported symptoms among seronegative participants requires discussion—possible explanations could include recall bias or other respiratory pathogens. Consider shortening subheadings and restructuring for clarity.

We thank the reviewer for these suggestions, and we have now included a more nuanced interpretation of our findings in the Discussion (lines 373-377). Possible explanations of our high symptom recall have been expanded in the limitation of our work (lines 263-264, 344-345, 347-349).

• Discussion and Interpretation:

Expand on the public health implications of findings: how should policymakers and global health actors use this evidence to improve inclusion of FSWs in surveillance and vaccination programs? Provide a deeper explanation of why expected risk differentials were not observed. End the discussion with a forward-looking paragraph linking lessons learned to pandemic preparedness, equity, and surveillance integration for key populations.

This is an excellent suggestion. We have now expanded our Discussion to include dialogue on the public health implications of our findings (lines 404-414).

• Limitations:

Strengthen the limitations section by acknowledging: possible misclassification due to antibody waning or sensitivity variation; recall bias from self-reported symptoms; and limited generalizability beyond Nairobi FSWs.

These factors have been added to our limitations section (lines 388-395).

Reviewer #2:

Reviewers report:

This manuscript describes a longitudinal seroepidemiological study of SARS-CoV-2 among female sex workers (FSWs) in Nairobi, Kenya. The authors report a high seroincidence early in the pandemic and assess associations between infection and socio-behavioural factors. The study addresses a relevant, under-researched population and adds important epidemiologic data. This is a well written manuscript, even though substantial improvements are needed in the justification of methods, transparency of statistical reporting, clarity of definitions, and compliance with ethical/data standards. Addressing these issues will significantly strengthen the manuscript.

We appreciate the time taken by the reviewer to give us such a thorough review.

Major Issues

1. Causal Inference and Confounding

The analysis identifies no behavioural or demographic associations with infection. I am somewhat surprised by this. But on further thinking, the observational design and limited adjustment for confounders (e.g., unmeasured non-sex work exposures, social and household mixing) surely undermine causal interpretations. This is a limitation. The authors should clearly state limitations regarding confounding and the inability to make causal inferences.

We have updated our limitations sections to address confounding and the inability to make causal inferences per the reviewer’s suggestions (lines 373-377 and lines 395-397).

2. Serological Testing Limitations

Reliance solely on serological assays, without PCR confirmation, limits specificity for incident infection and can introduce uncertainty regarding timing. During the pandemic, serological tests were often notoriously inadequate. The manuscript should more robustly justify the serology methodology, when PCR tests were widely available, and point out this specific limitation in detecting acute or recent infections.

Serological testing was chosen for practical reasons. Most importantly, we received feedback from the community that they would not participate in a study that involved mandatory PCR testing, as there was government-mandated quarantine and so a positive test would have major financial implications. We have now formally addressed this in our methods (lines 137-141) and limitations (line 388-389).

3. Statistical Methods and Power

While the manuscript references post-hoc corrections for multiple testing, reporting lacks detail and results tables do not always clarify which findings remained robust after correction. Null results are not accompanied by formal sensitivity or power analyses, raising concerns about undetected moderate associations. Is there something that can be done about that?

We thank the reviewer for raising these concerns. We have added clarifying details to our Tables 3-5 to make it clear that p-values were unadjusted, and we have reworded our statistical methods to make the exploratory nature of our analysis more apparent to readers (lines 173-174, 370-371).

Although the reviewer is correct that weaker associations may have been missed, a sample size of 1000 is not trivial. We have now addressed concerns about undetected moderate associations within our limitations section (lines 398-399).

4. Ethical and Data Availability Compliance

The current data availability statement (“upon request”) does not fully comply with PLOS ONE’s data sharing policy. What are the specific data sharing and repository plans that the authors have. Or at least could you transparently state justified restrictions?

Data availability statement has been updated to address the data sharing restriction unique to our study.

I also found some minor Issues that could be addressed to improve this manuscript.

1. Definitions and Clarity

The distinction between “seroincident” and “seroprevalent” cases, and categorization of symptomatic disease, are operationally vague or inconsistently described. More precise definitions are needed for reproducibility. To be honest, I am not so familiar with “sero-incident’.

We appreciate the concerns raised about use of “seroincident” and “seroprevalent”. The terms are now used more carefully throughout the manuscript, and we hope that this is now more clear.

2. Tables and Supplementary Data

Several tables reference ambiguous or unclear variable names (e.g., “TITLE of sleeping rooms in home mean”). All table and variable names should be reviewed for clarity.

All tables have been reviewed for clarity, in refences to the example variable we have changed the “#” symbol to “number” to avoid any confusion in Tables 1, 3-5.

3. Contextualization and References

Contextualization could be improved by citing broader literature on COVID-19 risk in key populations, particularly FSWs in low- and middle-income countries.

We had now added several references that expand on COVID-19 risk in FSWs in our Introduction (lines 79-87)

4. Language and Copyediting

The manuscript contains minor typographical and language errors, with some awkward phrasing. A final round of careful editing will enhance clarity and readability.

We thank the reviewer for this suggestion. We have done a thorough round of editing on the entirety of the manuscript to improve readability and try to reduce awkward phrasing (various lines)

Recommendation

Major revision

Reviewer #3:

This is a well written and straightforward manuscript characterizing the seroincidence of SARS-CoV-2 infection early in the COVID-19 pandemic in a cohort of female sex workers in Nairobi, Kenya. The manuscript is clearly written and provides data that adds to the published literature describing the high seroincidence and low symptomatic rate of SARS-CoV-2 infection in Sub-Saharan Africa during the pandemic. Surprisingly, the manuscript did not identify any behavioral or demographic factors associated with seroincidence in this cohort.

There are a few revisions that would improve the paper:

• The authors report that the seroincidence of 27.7% in this FSW cohort was not higher than the general population - which they cite as increasing from 4.3% in 2020 to 48.5% in 2021. However, the sample collection for this FSW cohort was limited to followup samples collected June 2020-February 2021, and it needs to be a bit more clearly stated in the intro and discussion the exact timing of the seroincidence rates of other populations they are comparing to – i.e. what month in 2021 does the seroincidence rate reflect in the other populations (without asking the reader to look at the references).

We thank the reviewer for this comment and have made changes to both our Introduction and Discussion to make more concise comparisons of seroincidence rates between our cohort and other population at the same time point (lines 65,69, 330-332).

• Baseline samples were collected between June-Dec 2019. Follow up samples that were tested were collected between June 2020-February 2021, a 7-month window. The authors note the mean of 189 days between start of pandemic and follow-up visit date in the text and at the bottom of Table 3. This would be better if it was a median and regardless of median or mean – the range needs to also be reported in both the text and in Table 3 - and the authors should more clearly state the caveat that some individuals were only followed for a few months, and thus the seroincidence reported is inevitably an underestimate of the true seroincidence in this cohort by Feb 2021. The data would be more accurate if there was a sample from everyone from Jan-Feb 2021, but this is clearly not possible at this point, so the limitation should be clearly stated, and the data in the supplemental table should described in the resutls section and be moved to the main text to show the higher rate in the group sampled later.

The median of follow-up visit dates is now reported, instead of mean, in Table 3 as well as in text (lines 35, 223).

We have added language in the limitations section of this manuscript that states possible underestimation of true seroincidence due to short follow up periods (lines 390-392).

Additionally, as suggested by the reviewer, we have moved the supplemental table into the main text and results section (lines 233-242) (Table 4).

• This reviewer appreciates the use of more than one antigen to define seropositive status and the reporting of the false-positives with only 1 antigen detected. Would be good to comment on this in the discussion noting that RBD had the lowest background - and also the reasons that only 2 of the 3 antigens are detected in ~10% of samples.

As suggested by the reviewer, we have expanded our Discussion section to address the false-positive rate and the low background levels of RBD antibodies (lines 378-387), as well as the fact that baseline presence of antibodies against one antigen was not protective against subsequent infection.

• However, the authors then use the single-positives as a measure of those with pre-pandemic antibodies to SARS-CoV-2 as stated on Page 13 and Table 4. If they are suggesting that these are false positives due to the presence of cross-reactive antibodies to other infections (possibly related coronaviruses, etc..) then they should state that as such. Otherwise it seems odd to use the false positives from an assay to measure the potential of pre-existing immunity.

False-positives in our pre-pandemic

Attachment Submitted filename: Response to Reviewers.docx

# Peer review: Decision Letter 1

**DOI:** 10.1371/journal.pone.0327692.r003

**Authors:** Leonard Uzairue, Leonard Uzairue

27 May 2026

SARS-CoV-2 infection in female sex workers from Nairobi, Kenya early in the COVID-19 pandemic: seroincidence and behavioural associations.

PONE-D-25-32697R1

Dear Dr. Yang,

We’re pleased to inform you that your manuscript has been judged scientifically suitable for publication and will be formally accepted for publication once it meets all outstanding technical requirements.

Within one week, you’ll receive an e-mail detailing the required amendments. When these have been addressed, you’ll receive a formal acceptance letter and your manuscript will be scheduled for publication.

An invoice will be generated when your article is formally accepted. Please note, if your institution has a publishing partnership with PLOS and your article meets the relevant criteria, all or part of your publication costs will be covered. Please make sure your user information is up-to-date by logging into Editorial Manager at Editorial Manager® and clicking the ‘Update My Information' link at the top of the page. For questions related to billing, please contact billing support.

If your institution or institutions have a press office, please notify them about your upcoming paper to help maximize its impact. If they’ll be preparing press materials, please inform our press team as soon as possible -- no later than 48 hours after receiving the formal acceptance. Your manuscript will remain under strict press embargo until 2 pm Eastern Time on the date of publication. For more information, please contact onepress@plos.org.

Kind regards,

Leonard Ighodalo Uzairue, PhD

Academic Editor

PLOS One

Additional Editor Comments (optional):

Reviewers' comments:

Reviewer's Responses to Questions

**Comments to the Author**

1. If the authors have adequately addressed your comments raised in a previous round of review and you feel that this manuscript is now acceptable for publication, you may indicate that here to bypass the “Comments to the Author” section, enter your conflict of interest statement in the “Confidential to Editor” section, and submit your "Accept" recommendation.

Reviewer #2: All comments have been addressed

**********

2. Is the manuscript technically sound, and do the data support the conclusions?

The manuscript must describe a technically sound piece of scientific research with data that supports the conclusions. Experiments must have been conducted rigorously, with appropriate controls, replication, and sample sizes. The conclusions must be drawn appropriately based on the data presented.

Reviewer #2: Yes

**********

3. Has the statistical analysis been performed appropriately and rigorously?

Reviewer #2: Yes

**********

4. Have the authors made all data underlying the findings in their manuscript fully available?

The PLOS Data policy requires authors to make all data underlying the findings described in their manuscript fully available without restriction, with rare exception (please refer to the Data Availability Statement in the manuscript PDF file). The data should be provided as part of the manuscript or its supporting information, or deposited to a public repository. For example, in addition to summary statistics, the data points behind means, medians and variance measures should be available. If there are restrictions on publicly sharing data—e.g. participant privacy or use of data from a third party—those must be specified.

Reviewer #2: Yes

**********

5. Is the manuscript presented in an intelligible fashion and written in standard English?

PLOS ONE does not copyedit accepted manuscripts, so the language in submitted articles must be clear, correct, and unambiguous. Any typographical or grammatical errors should be corrected at revision, so please note any specific errors here.

Reviewer #2: Yes

**********

6. Review Comments to the Author

Please use the space provided to explain your answers to the questions above. You may also include additional comments for the author, including concerns about dual publication, research ethics, or publication ethics. (Please upload your review as an attachment if it exceeds 20,000 characters)

Reviewer #2: The recommended revisions have been adequately addressed. The typos and grammar have also been sufficiently corrected. This is an improved version. I have no further corrections to request.

**********

7. PLOS authors have the option to publish the peer review history of their article (what does this mean?). If published, this will include your full peer review and any attached files.

If you choose “no”, your identity will remain anonymous but your review may still be made public.

**Do you want your identity to be public for this peer review?**  For information about this choice, including consent withdrawal, please see our Privacy Policy.

Reviewer #2: **Yes:**  Matilu Mwau

**********

# Editor report: Acceptance letter

**DOI:** 10.1371/journal.pone.0327692.r004

**Authors:** Leonard Uzairue, Leonard Uzairue

PONE-D-25-32697R1

PLOS One

Dear Dr. Yang,

I'm pleased to inform you that your manuscript has been deemed suitable for publication in PLOS One. Congratulations! Your manuscript is now being handed over to our production team.

At this stage, our production department will prepare your paper for publication. This includes ensuring the following:

* All references, tables, and figures are properly cited

* All relevant supporting information is included in the manuscript submission,

* There are no issues that prevent the paper from being properly typeset

You will receive further instructions from the production team, including instructions on how to review your proof when it is ready. Please keep in mind that we are working through a large volume of accepted articles, so please give us a few days to review your paper and let you know the next and final steps.

Lastly, if your institution or institutions have a press office, please let them know about your upcoming paper now to help maximize its impact. If they'll be preparing press materials, please inform our press team within the next 48 hours. Your manuscript will remain under strict press embargo until 2 pm Eastern Time on the date of publication. For more information, please contact onepress@plos.org.

You will receive an invoice from PLOS for your publication fee after your manuscript has reached the completed accept phase. If you receive an email requesting payment before acceptance or for any other service, this may be a phishing scheme. Learn how to identify phishing emails and protect your accounts at https://explore.plos.org/phishing.

If we can help with anything else, please email us at customercare@plos.org.

Thank you for submitting your work to PLOS ONE and supporting open access.

Kind regards,

PLOS ONE Editorial Office Staff

on behalf of

Dr. Leonard Ighodalo Uzairue

Academic Editor

PLOS One
